# Evidence of a hydraulically challenging reach serving as a barrier for the upstream migration of infection-burdened adult steelhead

**DOI:** 10.1093/conphys/coz023

**Published:** 2019-06-06

**Authors:** W M Twardek, J M Chapman, K M Miller, M C Beere, S Li, K H Kaukinen, A J Danylchuk, S J Cooke

**Affiliations:** 1 Fish Ecology and Conservation Physiology Laboratory, Department of Biology and Institute of Environmental and Interdisciplinary Science, Carleton University, Colonel By Dr., Ottawa, ON, Canada; 2 Fisheries and Oceans Canada, Pacific Biological Station, Hammond Bay Rd, Nanaimo, BC, Canada; 3 British Columbia Ministry of Forests, Lands, Natural Resource Operations and Rural Development, Fisheries Branch, Alfred Ave, Smithers, BC, Canada; 4 Department of Environmental Conservation, University of Massachusetts Amherst, Holdsworth Way, Amherst, MA, USA

**Keywords:** Adult, disease, microbes, rainbow trout, salmon, spawning

## Abstract

Anadromous fishes such as steelhead trout, *Oncorhynchus mykiss*, are exposed to a suite of infectious agents and migratory challenges during their freshwater migrations. We assessed infectious agent load and richness and immune system gene expression in gill tissue of Bulkley River (British Columbia, CA) steelhead captured at and upstream of a migratory barrier to evaluate whether infectious burdens impacted migration success. We further considered the potential influences of water temperature, sex and fish size on host infectious agents and transcription profiles. There were eight infectious agents detected in steelhead gill tissue, with high prevalence of the bacteria *Candidatus Branchiomonas cysticola* (80%) and *Flavobacterium psychrophilum* (95%) and the microparasite *Sphaerothecum destruens* (53%). Fish sampled at the falls had significantly greater relative loads of *Ca. B. cysticola* and *F. psychrophilum*, higher infectious agent richness and differential gene expression compared to fish captured upstream. *Flavobacterium psychrophilum* was only associated with immune gene expression (particularly humoral immunity) of fish sampled at the falls, while water temperature was positively correlated with genes involved in the complement system, metabolic stress and oxidative stress for fish captured upstream. This work highlights interesting differences in agent–host interactions across fisheries and suggests that hydraulic barriers may reduce the passage of fish with the heaviest infectious agent burdens, emphasizing the selective role of areas of difficult passage. Further, this work expands our knowledge of infectious agent prevalence in wild salmonids and provides insight into the relationships between infectious agents and host physiology.

## Teaser

Lay summary:

Differences in infectious agents and host immune gene expression were evaluated in the gill tissue of steelhead captured at and above a major hydraulic barrier on the Bulkley River. Fish sampled at the barrier had significantly greater abundance and diversity of infectious agents and differential gene expression.

## Introduction

Infectious agents (pathogenic microparasites including viruses, bacteria, fungi and protozoa) are widespread in aquatic environments (Price, 1980) and play an integral role in shaping aquatic ecosystems through disease processes (e.g. Cotner and Biddanda, 2002; Suttle, 2005; Smith, 2007). Infectious diseases have resulted in declines in some wild fish populations (Marty et al., 2003; Steinbach Elwell et al., 2009) and have been hypothesized as a factor contributing to collapse in others (Pearson et al., 1999; Gibson-Reinemer et al., 2017). Infectious agents in an active state induce changes to host physiology that can reduce performance of fish in the wild (i.e. disease; Jeffries et al., 2014a; Miller et al., 2014; Teffer et al., 2017; Bass et al., 2019). Fish have evolved immune mechanisms to protect against and reduce the cost of disease (Uribe et al., 2011), though ultimately there are fitness consequences associated with host-immune responses regardless of disease development (Zuk and Stoehr, 2002). Consequently, understanding infection dynamics and host physiological response in wild fish populations is important to provide context for monitoring any population scale changes. The study of infectious agent dynamics in wild fish has been limited due to challenges associated with data collection and the large spatial scale of these processes (Peeler and Taylor, 2011). Recent advances in molecular genetics (Lawson Handley, 2015) have allowed for rapid and cost-effective quantification of gene expression, including the use of high-throughput quantitative reverse transcriptase polymerase chain reactions (PCR) on the BioMark™ microfluidics platform, which has recently been validated for detection and quantification of salmon infectious agents (Miller et al., 2016) and detection of a molecular signature indicative of viral disease (Miller et al., 2017). This platform can help deduce when an infectious agent has moved from a carrier state to actively replicate and cause damage to the host, based on the host-immune response (Casadevall and Pirofski, 1999; Miller et al., 2014).

Infectious agent communities are dynamic and their prevalence among and within wild fish populations are highly dependent on both biotic and abiotic factors. Intrinsic biological differences of potential host species such as life-history stage (Castro et al., 2015), size (Boerlage et al., 2011; Molina-Fernandez et al., 2015), age (Marty et al., 2003), condition (Rohlenová et al., 2011) and sex (Richards et al., 2010; Teffer et al., 2017) may alter individual susceptibility to infection. For example, infectious hematopoietic necrosis virus causes significant mortalities in juvenile salmonids but is relatively nonpathogenic in adult fish (Traxler et al., 1997). Further, sexually mature female rainbow trout, *Oncorhynchus mykiss* (Linnaeus), have demonstrated greater parasitaemia and mortality from the blood parasite *Cryptobia salmositica* than sexually mature males (Currie and Woo, 2007). Environmental conditions may affect infectious agent virulence and host vulnerability to these organisms (Wedemeyer, 1970; Rottmann et al., 1992; Marcos-Lopez et al., 2010; Sepahi et al., 2013). Temperature, in particular, has a critical influence on infectious agent-fish host infection dynamics given the governing role the environment has on fish physiology (Brett, 1971; Fry, 1971; Yada and Tort, 2016) and infectious agent productivity (e.g. Thoen et al., 2016). Understanding the factors that contribute to infectious agent prevalence and host physiology is exceptionally important for anadromous fish that undertake challenging, long-distance migrations and experience a suite of physiological changes and novel agents upon returning to freshwater to complete their reproductive cycle (Cooke et al., 2011; Miller et al., 2014).

Migratory Pacific salmonid species typically enter rivers to spawn after completing the marine portion of their lifecycle (Schaffer, 2004). Pacific salmonids have been in decline across much of their distribution (Beamish et al., 1999) and there have been notable shifts in survival of return migrating adult salmon (Cooke et al., 2006; Hinch et al., 2012). Research has highlighted several potential sources of prespawn mortality including increasing water temperatures (Hinch et al., 2012; Bowerman et al., 2018), fisheries captures (Baker and Schindler, 2009) and infectious agents (Traxler et al., 1998). The impacts of which may be exacerbated in areas of difficult passage (Hinch and Bratty, 2000).

Steelhead *O. mykiss* (Walbaum) are unique to other Pacific salmonids in their potential iteroparity and overwintering behaviour prior to spawning (Scott and Crossman, 1973). During their spawning migrations, steelhead may remain in rivers for up to 10 months, increasing their potential exposure to accumulated thermal units and to infectious agents relative to other anadromous salmonids. Steelhead provide an ideal model to study the impact of infectious agents on migratory fish because their condition does not decline as rapidly and severely during migrations relative to semelparous salmon that undergo natural senescence. Despite growing evidence that infectious agents can have negative impacts on wild salmon populations (Jeffries et al., 2014a; Teffer et al., 2017; Bass et al., 2019), the influence of infectious agents on wild steelhead remains relatively unexamined. The Pacific Northwest maintains some of the last remaining entirely wild steelhead runs on Earth, with the rivers of the Skeena watershed, British Columbia, supporting many of these wild populations.

Adult steelhead returning to spawn in the Bulkley River of British Columbia, Canada, must undertake a physiologically challenging migration through Witset Falls 314 km into their migration. Fish can be observed holding at the base of the falls before making their ascent to spawning grounds. The Wet’suwet’en First Nation have a long-standing salmon fishery where they dip net fish holding in eddies as they attempt to ascend the falls. They also conduct a steelhead mark-recapture program in collaboration with the British Columbia Ministry of Forests, Lands, Natural Resource Operations and Rural Development—Skeena Region, with the recapture occurring part way up the falls. Above the falls, there is a high-effort catch-and-release recreational angling fishery.

To determine if the falls act as a migration barrier for weaker fish we evaluated the condition of steelhead based on the transcription of biomarkers known to be associated with immunity, stress, osmoregulation and metabolism, as well as infectious agent presence and relative load at and above this natural barrier to migration. We hypothesized that infectious agent load would impact fish migration success (i.e. highly infected fish would have a low success rate at passing the migration barrier). We also explored relationships between the relative loads of infectious agents and host biomarkers and predicted that differential immune stimulation would be associated with a subset of infectious agents with the greatest pathogenic potential. We further considered the influence of water temperature, sex and fish size on host biomarkers and infectious agent load and richness. We predicted that host biomarkers of immunity and stress would be generally upregulated with warmer temperatures, as observed in previous temperature challenge studies (Jeffries et al., 2012a, 2014b) and would be greater for females, as they have shown higher stress and prespawn mortality during spawning migrations (Kubokawa et al., 1999; Teffer et al., 2017).

## Methods

### Study site and collection

Adult steelhead were captured from 23 September to 29 October 2016 by dip net and angling and nonlethally sampled for gill tissue. All fish captured by dip net were from Witset Falls (314 rkm; height, 15 m) by Wet’suwet’en fishers. Steelhead captured by dip net were transferred in <5 s to a transport sling, which a runner then transported to a water-filled sampling trough (total air exposure of <25 s). Angling was completed upstream of Witset Falls (325–407 rkm) by local or guided anglers that agreed to assist with the study. Anglers used a combination of fly fishing, spin fishing and centre pin fishing and various sizes of flies, inline spinners and artificial worms. Fish were transferred directly into a sampling trough positioned in the river upon landing (no air exposure period exceeded 30 s). The date of sampling differed between capture methods, which represented inherent differences in the fisheries we worked with to sample migrating steelhead. The Wet’suwet’en salmon fishery operates from early August until the last week in September at Witset Falls while salmon and steelhead are actively migrating. The recreational fishery operates primarily between mid-September to early November and is typically completed upstream of the falls where there is greater and safer access to the river. Steelhead anglers almost exclusively target slow moving water or refuge sites where steelhead are known to hold prior to overwintering in the river (Keefer et al., 2009). These sampling groups therefore reflect fish in two different stages of migration and freshwater acclimation, which has been previously confirmed by telemetry (Twardek et al., 2019).

Once landed and secured in the water-filled sampling trough (or recovery bag), steelhead were sampled nonlethally for ~ 0.5 mg of gill tissue (2–3 filament tips) using sterilized diagonal cutting pliers to prevent tearing (see Cooke et al., 2005 for details on the technique). The presence of infectious agents and corresponding physiological changes can be effectively detected in gill tissue even when it is not the primary infectious tissue (Mendonca and Arkush, 2004; Tavares et al., 2016; Miller et al., 2017; Teffer et al., 2017) and many studies investigating infection status have used this method (e.g. Cornwell et al., 2013; Teffer et al., 2017; Bass et al., 2019). Gill tissue was stored in 1.2 ml of RNAlater® solution (Qiagen, MD, USA) in 1.5 ml microtubes and stored at −20°C for future genomic analyses. Nonlethal gill biopsy sampling was undertaken due to conservation concerns for Bulkley steelhead. All steelhead were assessed for fish length (FL; mm) and sex and were sampled and released within 30 min of capture, during which they remained submerged in water. Surface water temperatures (°C) at the point of capture were taken using a handheld digital thermometer (Taylor Precision Digital Thermometer, #9847, Taylor USA, Oak Brook, IL, USA) as an indication of the broader scale patterns in temperatures experienced by fish throughout the river and season. All sampling was conducted in accordance with the guidelines of the Canadian Council on Animal Care as administered by Carleton University (protocol 106247).

### Sample analyses

All sample analyses were completed at the Pacific Biological Station (Fisheries and Oceans Canada). To determine the abundance of infectious agents and host gene expression simultaneously, high-throughput nanofluidic quantitative PCR (qPCR; Fluidigm BioMark™ Dynamic Array, CA, USA; outlined in Jeffries et al., 2014a and Miller et al., 2014, 2016) were undertaken on gill RNA using assays for 47 infectious agents known or suspected to cause disease in salmon (Supplementary Table S1) and 59 genes with a broad range of immune pathways of defence and stress responses. The magnitude of host response (measured herein via host gene expression) can be used to evaluate the relative likelihood an infectious agent is causing damage and therefore disease to the host (Casadevall and Pirofski, 1999; Miller et al., 2014, 2017). To provide an overview of the processing method, each sample was independently homogenized with a MM301 mixer mill (Restch Inc., PA, USA) and aliquots of the aqueous phase (containing genetic material) were taken. RNA quantity (A260) and purity (A260/280) were evaluated by spectrophotometry, and samples were normalized accordingly. RNA was used to construct cDNA (Invitrogen™ SuperScript™ VILO™ CA, USA, cDNA Synthesis Kit) that underwent a specific targeted amplification (STA) to provide sufficient template molecules for qPCR on the Fluidigm BioMark (impacts of STA on the analytical performance of infectious agent assays presented in Miller et al., 2016). Samples were then treated with ExoSAP-IT® PCR Product Cleanup (MJS BioLynx Inc., ON, Canada) to remove unincorporated nucleotides and primers and were diluted 5-fold with DNA Suspension Buffer (Teknova, Hollister, California). Sample and assay mixes were loaded onto the Fluidigm Dynamic Array using an IFC Controller HX, and the GE 96 × 96 Standard v1.pcl. (TaqMan®) protocol was used for qPCR. TaqMan assays to infectious agents were designed to target RNA, which can assess microparasites in an active state through detection of mRNA, and allows for simultaneous screening of RNA viruses (Miller et al., 2016) and host genes (Miller et al., 2017). A subset of samples with strong activation of a biomarker panel associated with a viral disease development (VDD) state (Miller et al., 2017), but that did not test positively for any viruses in our initial panel, was screened for 10 additional salmon viruses recently discovered in BC salmon (Gideon Mordecai, unpublished data). On each dynamic array, we included a negative extraction control, a negative and positive cDNA control, a negative and positive control during the STA stage and a control that did not undergo pre-amplification (as described in Miller et al., 2016).

Samples and agent assays were run in duplicate during the final qPCR resulting in quadruplicate measures of infectious agent expression while biomarker assays and reference assays (78d16.1, MrpL40 and Coil-P84) were run singly, resulting in duplicate cycle threshold (CT) values for each sample. The BioMark real-time PCR analysis software was used to assign CT values for each reaction curve based on visual assessment of curve shape and replicate similarity (a negative scale from 0–40 corresponding to the maximum number of cycles completed on the BioMark). CTs were averaged for host biomarker and infectious agent assays. Samples that had two or more quadruplicates fail to amplify infectious agent products were treated as negative detections. Positive infectious agent detections were only assigned for samples with a CT below the 95% limit of detection, which varied for each infectious agent assay (Miller et al., 2016). Averaged CT scores underwent an efficiency correction using the formula CT((log(1 + efficiency)/(log(2))) as specified by the GenEx Software (www.multid.se). Biomarker assays with efficiencies outside 1.0 ± 0.2 were considered failed and were removed from analysis (Supplementary Table S2). Biomarker data were normalized against reference genes (developed in-house by the Molecular Genetics Laboratory at the Pacific Biological Station, Nanaimo, BC) and pooled sample using the 2^ΔΔCT method (Livak and Schmittgen, 2001; Teffer et al., 2017).

### Statistical analyses

Prior to analysis, infectious agent abundance data were transformed to a positive scale by subtracting each CT value from 40 (the maximum number of cycles completed on the BioMark as per Bass et al., 2017). This measurement reflects the relative abundance or ‘loading’ for each microbe between individuals and is hereby termed ‘relative load’. The variable ‘agent richness’ was also calculated from infectious agent data and was defined as the number of individual infectious agents found within each fish.

To test the relationship between the relative loads of infectious agents and the predictor variables of sampling location, water temperature at the time of sampling, sex and fish size, we used multiple regression models. Two separate multiple regression models used the relative loads of either *Flavobacterium psychrophilum* or *Candidatus Branchiomonas cysticola* as response variables, limiting the data to positive infectious agent detections only. Only these two agents were present in enough fish to merit analysis of effect. Diagnostic plots suggested that the relative load models for both infectious agents had minimal deviations from normality. To test the relationship between infectious agent richness and the predictor variables of sampling location, water temperature at the time of sampling, sex and fish size, we used a Poisson regression model to account for count data and a right skewed distribution. To evaluate the relationship between relative loads of *Ca. B. cysticola, F. psychrophilum* and *Sphaerothecum destruens* we used Spearman’s ran correlations for all individuals with positive detections.

Kruskal’s nonmetric multidimensional scaling (NMDS) was used to evaluate relationships between sampling location and host gene expression values. NMDS is an ordination technique that uses nonlinear dimensionality reduction to visualize relationships between observations for multiple response variables and is an effective statistical approach for gene expression data (Taguchi and Oono 2005). A Bray–Curtis distance matrix was created based on the gene expression values for each fish (R function ‘metaMDS’; package ‘vegan’). Dimensionality of the ordination was determined as the fewest number of axes needed to reduce the disagreement between rank orders of observed and predicted distances to appropriate levels (stress < 0.2; Kruskal, 1964). Biomarker scores were determined using weighted averages of all predicted distances. The external variable sampling location was fit into the ordination using the ‘envfit’ function (package ‘vegan’) to test for differences in gene expression above and at the falls. The envfit function fits external variables to predicted points by maximizing their correlation. Separate NMDS analyses for fish sampled at the falls and above the falls were completed to determine whether infectious agents and other external variables were having unique impacts on fish physiology at different stages in the migration. These two NMDS analyses were completed for fish at and above the falls fitting the relative loads of *F. psychrophilum* and *Ca. B cysticola*, agent richness, water temperature, sex and FL as external variables. Only infectious agents with high prevalence were fit to the ordination to reduce bias associated with agents that had only a few positive detections (as per Teffer et al., 2017). Continuous variables (agent loads, agent richness, water temperature and FLs) were plotted as vectors that represent maximal correlation of each variable with the ordination. Vector lengths were shortened and biomarker labels were adjusted to increase plot comprehension. Significance was assessed at α < 0.05. Where applicable, means and standard errors are presented. All statistical analyses were conducted in R Version 3.4.3 (R Core Team, 2015), and figures were constructed in Sigma Plot Version 11.0.0.75.

**Table 1:** The prevalence and relative loads (40-CT) of infectious agents present in adult Bulkley River steelhead across fisheries/sampling locations.

**Assay name**	**Agent name**	**Type**	**Prevalence (%)**		**Relative load (40-CT)**	
			**At falls**	**Above falls**	**At falls**	**Above falls**
c_b_cys	*Candidatus Branchiomonas cysticola*	Bacterium	93	80	27.6 (22.4–32.1)	23.9 (15.9–31.7)
fl_psy	*Flavobacterium psychrophilum*	Bacterium	100	92	22.6 (18.7–24.2)	18.0 (13.2–23.6)
pch_sal	*Piscichlamydia salmonis*	Bacterium	13	4	22.7 (21.7–23.8)	13.1
lo_sal	*Loma salmonae*	Parasite	20	0	15.8 (14.7–16.7)	0
pa_ther	*Paranucleospora theridion*	Parasite	13	0	17.1 (15.0–19.3)	0
te_bry	*Tetracapsuloides bryosalmonae*	Parasite	0	4	0	24.7
sp_des	*Sphaerothecum destruens*	Parasite	66	40	21.4 (15.8–24.7)	19.1 (14.4–25.2)

Each assay lists the abbreviation, infectious agent name, type of agent, prevalence and mean relative load (range) by location. Mean relative loads were calculated using positive infectious agent detections only

**Figure 1:**
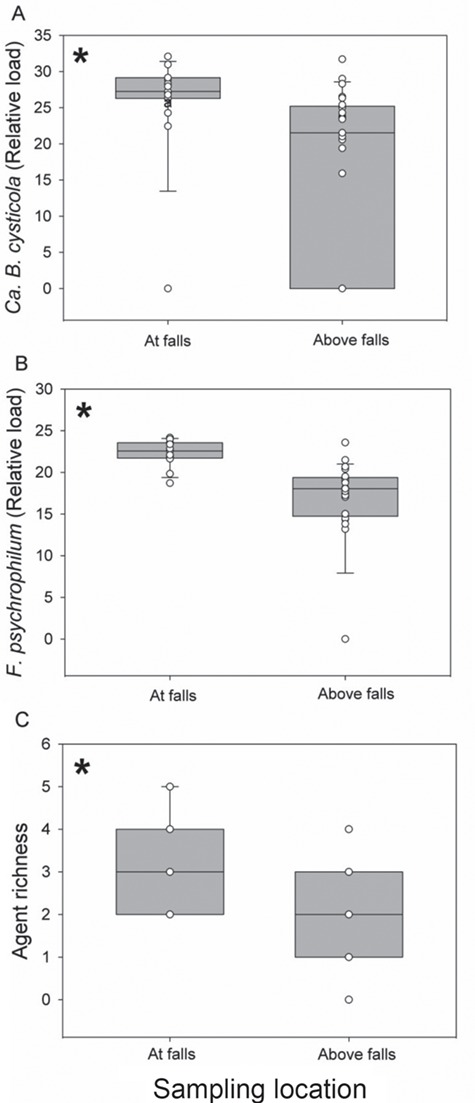
Boxplots depicting the relative load of (**A**) *Ca. B. cysticola* (**B**) *F. psychrophilum* and (**C**) agent richness in the gill tissue of steelhead sampled above the falls and at the falls. Asterisks denote a statistically significant difference (*P* < 0.05).

## Results

Fifteen steelhead (598 ± 62 mm; 53% female) were captured by dip net at Witset Falls (314 rkm) between 23 and 29 September 2016. An additional 25 steelhead (688 ± 16 mm; 68% female) were captured by angling (325–407 rkm) between 23 September and 29 October 2016. Sampling temperatures remained cool for fish both at (8.6 ± 0.4°C; 7.0–11.5°C) and above (6.2 ± 0.3°C; 3.0–10.3°C) the falls. Sampling temperature was closely correlated with date (*r* = −0.76; *P* < 0.01) highlighting its usefulness as a proxy for broad scale changes in temperatures experienced as the sampling period progressed.

### Infectious agents

Seven infectious agents were initially detected in Bulkley River steelhead gill tissue, though only two were sufficiently prevalent to evaluate drivers of their abundance and their influence on host physiology. *Candidatus Branchiomonas cysticola* (80%), *F. psychrophilum* (95%) and *S. destruens* (53%) were the most prevalent infectious agents, with low prevalence of *Loma salmonae* (20%), *Piscichlamydia salmonis* (13%), *Paranucleospora theridion* (13%) and *Tetracapsuloides bryosalmonae* (4%; Supplementary Table S1). The prevalence of all agents was higher in fish captured at the falls than above (Table 1), except for *T. bryosalmonae* that was only detected in one fish above the falls. The relative load of *Ca. B. cysticola* was not significantly correlated with water temperature at the time and location of sampling (*R*^2^ = 0.03; *P* = 0.25), sex (*P* = 0.97) or FL (*R*^2^ = 0.03; *P* = 0.33) but was significantly greater for fish sampled at the falls than above (*t* = 2.86; *P* < 0.01; Fig. 1A). The relative load of *F. psychrophilum* was not significantly correlated with water temperature (*R*^2^ = 0.13; *P* = 0.94), sex (*P* = 0.07) or FL (*R*^2^ = 0.01; *P* = 0.31) but was significantly greater for fish sampled at the falls than above (*t* = 4.37; *P* < 0.01; Fig. 1B; Table 2). The relative loads of *Ca. B. cysticola* and *F. psychrophilum* were not closely correlated (*r*_s_ = 0.28), neither were *Ca. B. cysticola* and *S. destruens* (*r*_s_ = 0.28) nor *F. psychrophilum* and *S. destruens* (*r*_s_ = 0.39). Agent richness was also significantly greater (χ^2^ = 11.79; DF = 1; *P* < 0.01) for fish sampled at the falls (3.1 ± 0.3 agents) compared to fish above (2.1 ± 0.2 agents; Fig. 1C; Table 3). Water temperature (*P* = 0.19), sex (*P* = 0.12) and FL (*P* = 0.94) were not significant predictors of agent richness.

### Host gene expression

**Figure 2:**
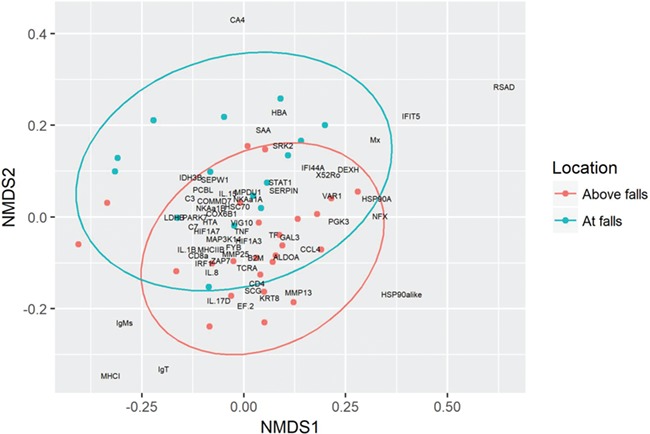
An NMDS plot of host gene expression in the gill tissues of Bulkley River steelhead (*n* = 40). The colour represents fishery/sampling location (red represents angling upstream of falls; blue, dip net at falls). Confidence ellipses (95%) are shown in blue and red. The most influential variables on NMDS1 are RSAD (8.7%), IFIT5 (5.6%) and HSP90alike (5.3%) and on NMDS2 are CA4 (6.9%), MHCI (5.9%) and IgT (5.7%).

Gene expression data were successfully reduced into a 2D ordination (stress, 0.18; Fig. 2). The genes most positive on NMDS1 included RSAD, IFIT5, Mx, NFX, DEXH, VAR1, X52Ro and IFI44A, which are indicative of a VDD response when co-expressed (Miller et al., 2017). Five individuals had a value >0.18 on NMDS1, suggesting they were in a state of VDD. Four of these individuals were captured above the falls and one was captured at the falls. Further screening of these samples with a more comprehensive panel of 10 viruses newly discovered through application of the VDD panel in BC salmon (Gideon Mordecai, unpublished data) revealed a novel strain of cutthroat trout virus (CTV-2) detected in two of the five samples (data not shown). The other three samples may have a yet to be identified virus inducing this viral disease signature.

Sampling location was significantly associated with the ordination gradient (*P* < 0.01). Fish sampled at the falls (earlier in migration during the active upstream movement phase) were closer in ordination space to osmoregulatory genes typically elevated during saltwater preparation (HBA and CA4). Fish sampled upstream of the falls (more advanced in migration) were closer in ordination space to genes related to stress and immunity such as IL-17D, IL8 and MMP13, particularly humoral immune genes (IgMs and IgT).

**Table 2:** Multiple regression output predicting the relative load of *Ca. B. cysticola* and the relative load of *F. psychrophilum* in the gill tissue of steelhead captured in the Bulkley River, BC.

**Variable**	**Relative load: *Ca. B. cysticola***			**Relative load: *F. psychrophilum***		
**Parameter**	**Estimate ± SE**	***t*-value**	***P***	**Estimate ± SE**	***t*-value**	***P***
(Intercept)	32.427 ± 6.124	5.295	<0.001	14.531 ± 3.431	4.235	<0.001
Fishery/location: at falls^**^	4.795 ± 1.676	2.861	0.008	4.066 ± 0.929	4.372	<0.001
Water temperature	−0.472 ± 0.399	−1.182	0.248	0.016 ± 0.224	0.071	0.943
Sex: male	0.053 ± 1.394	0.038	0.970	1.456 ± 0.786	1.852	0.073
FL	−0.008 ± 0.008	−1.001	0.326	0.004 ± 0.004	1.031	0.310

The model includes fisheries/sampling location and sex as categorical variables and water temperature (°C) and FL (mm) as continuous variables. Asterisks denote a statistically significant difference (*P* < 0.05)

**Table 3:** Poisson regression output predicting infectious agent richness in the gill tissue of steelhead captured in the Bulkley River, BC.

**Variable**	**Agent richness**		
**Parameter**	**χ** ^**2**^	**DF**	***P***
Fishery/location^**^	11.791	1	<0.001
Water temperature	1.739	1	0.187
Sex	2.511	1	0.113
FL	0.005	1	0.944

The model includes fishery/sampling location and sex as categorical variables and water temperature (°C) and FL (mm) as continuous variables. Asterisks denote a statistically significant difference (*P* < 0.05)

### Gene expression of fish captured by dip net at Witset Falls (earlier in migration)

**Figure 3:**
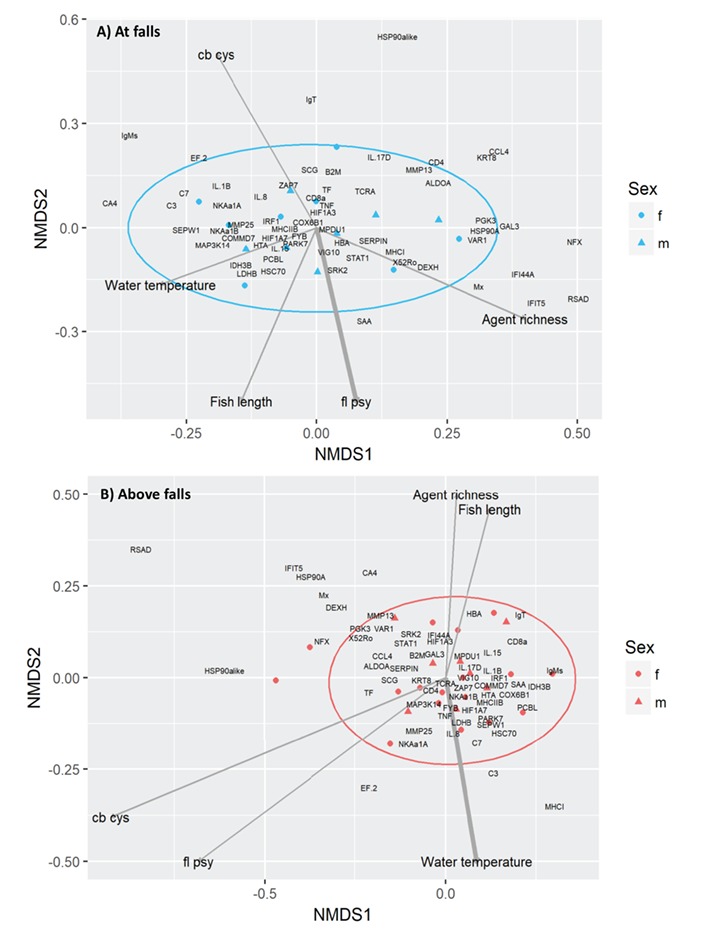
NMDS plots of host gene expression in the gill tissues of Bulkley River steelhead (*n* = 40) caught (**A**) at Witset Falls and (**B**) above Witset Falls. The shape of each point reflects sex (circle reflects female; triangle, male). Vectors represent external variables fit into the ordination including the relative loads of *Ca. B. cysticola* and *F. psychrophilum*, agent richness, water temperature and FL. Bolded lines indicate significant vectors. Confidence ellipses (95%) are shown in blue and red. In plot (A) the most influential variables on NMDS1 are RSAD (5.0%), NFX (4.9%) and IFIT5 (4.2%) and on NMDS2 are HSP90alike (8.6%), IgT (5.8%) and SAA (4.2%). In plot (B) the most influential variables on NMDS1 are RSAD (8.6%), HSP90alike (6.2%) and IFIT5 (4.3%) and on NMDS2 are RSAD (8.6%), MHCI (3.1%) and IFIT5 (4.3%).

Gene expression data were reduced to a 2D ordination (stress, 0.15) for the fish captured at Witset Falls (Fig. 3A). Relative load of *F. psychrophilum* was correlated with the ordination gradient (*R*^2^ = 0.52; *P* = 0.01) being slightly positive on NMDS1 and negative on NMDS2. Relative load of *F. psychrophilum* was close in ordination space to SAA and opposite to IgT. Neither the relative load of *Ca. B. cysticola* (*R*^2^ = 0.30; *P* = 0.13), infectious agent richness (*R*^2^ = 0.10; *P* = 0.51), water temperature (*R*^2^ = 0.03; *P* = 0.82), sex (*P* = 0.37) nor FL (*R*^2^ = 0.15; *P* = 0.51) were correlated with the ordination gradient.

### Gene expression of fish captured by angling upstream of Witset Falls (later in migration)

Gene expression data were reduced to a 2D ordination (stress, 0.16) for the fish captured above Witset Falls (Fig. 3B). Neither infectious agent load, *F. psychrophilum* (*R*^2^ = 0.09; *P* = 0.51) or *Ca. B. cysticola* (*R*^2^ = 0.15; *P* = 0.32), was correlated with the ordination gradient, nor was infectious agent richness (*R*^2^ = 0.04; *P* = 0.73). Water temperature had a significant relationship with the ordination gradient (*R*^2^ = 0.27; *P* = 0.03) and was close in ordination space to C3 and C7 and opposite to CA4. Neither sex (*P* = 0.73) nor FL (*R*^2^ = 0.05; *P* = 0.52) was correlated with the ordination gradient.

## Discussion

Wild steelhead captured by dip net below Witset Falls were found to have greater infectious agent richness, higher relative loads of *Ca. B. cysticola*, and *F. psychrophilum*, and differential gene expression compared to fish captured upstream of the falls by angling. We hypothesize that higher infectious burdens (in gill tissue) in fish sampled below the falls may have impacted the ability of fish to ascend the falls (i.e. migratory culling; Bradford et al., 2010; Altizer et al., 2011). Below, we discuss prominent differences between these groups of fish and how those differences may contribute to the observed findings. Fish sampled at Witset Falls were sampled earlier in the season and further downstream suggesting lower freshwater preparedness. This is consistent with the elevated expression of CA4 and HBA in this group of fish which are important in the earlier stages of freshwater acclimation (Houde et al., in review) and higher expression of humoral immune genes (IgMs and IgT) and B2M, MHCIIB and IRF1 that tend to increase over the course of migration in sockeye salmon (Teffer et al., 2017). Radio telemetry work has indicated that steelhead upstream of Witset Falls represent successful migrants that are holding at or near spawning sites, while fish at Witset Falls are in the active stage of upstream migration (Twardek et al., 2019; Økland et al., 2001).

### Sampling location—fisheries selectivity

Witset Falls may act as a natural barrier influencing infection dynamics during steelhead migration, and successful migrants (angled fish) may be biased towards fish with lower infectious agent loads relative to actively migrating fish at Witset Falls (i.e. migratory culling; Altizer et al., 2011; Risely et al., 2018). Witset Falls constitutes the most difficult hydraulic reach of the Bulkley River. Steelhead are known to delay their migration at this point, resulting in large densities of steelhead below the falls, and ~8–12% of steelhead that enter the canyon/falls area do not successfully migrate upstream of the falls (Welch et al., 2009; Twardek et al., 2019). Confinement of steelhead below this migratory barrier may increase exposure to infected individuals and correspondingly increase infectious agent prevalence and loads at this location (Bebak-Williams et al., 2002). Direct fish to fish transfer of infections has been observed for *Ca. B. cysticola* in presmolt Atlantic salmon, *Salmo salar* (Linnaeus), suggesting transfer between steelhead may also occur during confinement (Wiik-Nielsen et al., 2017). Increased agent loads (including *Ca. B. cysticola* and *F. psychrophilum*) can entail reduced swimming capabilities, lethargy, negative effects on movement and decreased survival (Schachte, 1983; Kent et al., 1989; Barber et al., 2000; Wagner et al., 2003; Risely et al., 2018), potentially explaining why fewer heavily burdened fish were observed upstream of the falls. Evidence to this effect comes both from the finding that *F. psychrophilum* was correlated with gene expression of fish at the falls but not those that successfully migrated above to holding sites, and the finding of three agents capable of causing gill disease primarily detected in fish at the falls. If these agents were negatively impacting the osmoregulatory or oxygen-carrying capacity of the host, or causing inflammation, we cannot discount the possibility that they may have impacted the ability of the fish to ascend the falls. A concurrent telemetry study evaluating steelhead migratory survival following angling suggested that infectious agent-attributed mortality during fall migration is low for steelhead captured upstream of Witset Falls (Twardek et al., 2018). This study found one mortality (1.5% of fish) that could not be readily explained by the capture event itself, which indicates that infectious agent-induced mortality is minimal for fish upstream of the falls (Twardek et al., 2018). The notion of migratory culling is partly supported by the described telemetry work (Twardek et al., 2018, 2019), but future telemetry studies should additionally include gill agent-transcriptome analyses such as performed herein.

As presented above, our data indicate that fish caught in the dip net fishery had higher infectious burdens and potentially greater pathological effects of *F. psychrophilum* than those caught upstream by angling. Previous research has highlighted that various fishing gears have sampling bias that may select for fish with phenotypes that make them more vulnerable to capture (Diaz Pauli et al., 2015; Philipp et al., 2015). The Wet’suwet’en dip net fishery at the falls takes advantage of congregations of fish resting within small eddies as they attempt to ascend the falls. It is possible that fish with greater infectious agent burdens spend more time resting in these eddies and may be more vulnerable to capture by this fishery. Conversely, angling has been suggested to capture fish with higher metabolic rates (Hessenauer et al., 2015), lower metabolic stress (Louison et al., 2017) and higher activity levels (Alós et al., 2012), which would likely be correlated with reduced vulnerability to infectious agents (Maule et al., 1989; Pickering and Pottinger, 1989). This is consistent with our finding that angled fish had lower expression of genes responsive to oxygen availability and metabolism and lower prevalence and relative loads of infectious agents. However, these sampling biases can be inconsistent across gears, species and environments making it difficult to develop generalizations (Hollins et al., 2018). There is also a small amount of capture-and-release mortality from the dip net fishery (<1% of the population) that could reduce the number of fish with high infectious burdens upstream of the falls (Twardek et al., 2019). Our data cannot evaluate the extent of fisheries selectivity towards fish with certain infectious agent profiles without downstream angled fish data for comparison, but we recognize that this selectivity may be occurring between gear types in our study. Future research should consider both possibilities of migratory culling at natural barriers, and infection-related sampling bias associated with angling vs. net capture, especially for other species/regions where fish are harvested and fisheries would have stronger selective pressure.

### Infectious agents Ca. B. cysticola and F. psychrophilum

Steelhead migrating up the Bulkley River had high-observed prevalence of the bacteria *Ca. B. cysticola* and *F. psychrophilum*. *Ca. B. cysticola* transmission has primarily been recorded in the marine environment, while *F. psychrophilum* is only transmitted in freshwater (Tucker et al., 2018). Both bacteria have been found at high prevalence in wild salmon across BC (Bass et al., 2017; Teffer et al., 2017). *Candidatus Branchiomonas cysticola* has been associated with proliferative gill formation, bacterial cysts in the gills, epithelial hyperplasia and subepithelial inflammation associated with bacterial inclusions (Toenshoff et al., 2012; Mitchell et al., 2013; Wiik-Nielsen et al., 2017). *Flavobacterium psychrophilum* is typically most prevalent and pathogenic at temperatures below 10°C (Holt, 1987) and is the causative agent for bacterial cold-water disease, characterized by tissue necrosis and eventual mortality (Starliper, 2011; Nematollahi et al., 2003). The low temperature of the Bulkley River during sampling (7.1 ± 0.3°C) may explain the high prevalence of *F. psychrophilum*. In the nearby Babine River (Skeena watershed) *Flavobacterium spp.* were the most prevalent agents identified in rainbow trout, *O. mykiss* (Walbaum), where temperatures were even cooler at the time of sampling (Wellband and Heath, 2013).

Although we observed higher prevalence and loads of *Ca. B. cysticola* at rather than above the falls, we failed to observe any significant associations between host relative loads of *Ca. B. cysticola* and gene expression. This is consistent with previous studies on Pacific Salmon that have failed to observe impacts of *Ca. B. cysticola* on host physiology in freshwater (Bass et al., 2017; Teffer et al., 2017), but the agent has been associated with inflammation in Chum salmon in the marine environment (Cook, 2018). *Flavobacterium psychrophilum* was associated with negative or neutral expression of most immune genes (e.g. CD8a, Il-8, MHCIIB, IgT and IgMs). The greatest downregulation was observed in genes involved in the humoral immune response (IgT and IgMs), which are known to be suppressed by *F. psychrophilum* (discussed in Barnes and Brown, 2011; Teffer et al., 2017). This relationship between loads of *F. psychrophilum* and downregulation of immune genes has also been observed in adult sockeye salmon, *Oncorhynchus nerka* (Teffer et al., 2017) and immune challenged wild rainbow trout responding to *Flavobacterium spp*. (TNF, IFN and CXC8; Wellband and Heath, 2013) suggesting there may be a relationship between immunosuppression and *F. psychrophilum* (Barnes and Brown, 2011). This association between host immunity and *F. psychrophilum* was only present for fish captured at Witset Falls, which suggests the agent was causing damage to these fish (i.e. disease; Casadevall and Pirofski, 1999). It could be that fish with heavy burdens of *F. psychrophilum* were less capable of surpassing the hydraulically challenging swim through Witset Falls related to this damage (Kent et al., 1989; Risely et al., 2018).

### Sphaerothecum destruens and low prevalence infectious agents

Approximately half of the Bulkley River steelhead tested positively for *S. destruens*, a eukaryotic generalist parasite that can infect fish in both freshwater and marine ecosystems (Kent, 2011; Andreou and Gozlan, 2016). *Sphaerothecum destruens* has resulted in sublethal consequences for infected cyprinids including inflammation, lesions and cell death (Andreou et al., 2011). *Sphaerothecum destruens* was also associated with mortality in winter-run juvenile chinook salmon, *Oncorhynchus tshawytscha* (Walbaum), from the Sacramento River, though juvenile rainbow trout seemed to be more resilient to infection (Arkush et al., 1998). Three other infectious agents known to cause gill disease and associated mortality were observed primarily in fish caught in the dip net fishery at the falls: *P. theridion, P. salmonis* and *L. salmonae* (Nowak and LaPatra, 2006; Nylund et al., 2010, 2011; Schmidt-Posthaus et al., 2012; Speare and Lovy, 2012)*.* However, as these agents were not highly prevalent, we did not attempt to quantify their influence on host gene expression. Given previously reported consequences of these agents, future studies with larger sample sizes should consider the influence that they may have on salmonid host gene expression. Further research is also needed to understand the transmission requirements for these agents though it is known that *L. salmonae* may be transmitted in both marine and freshwater systems (Tucker et al., 2018).

Patterns in the expression of a suite of genes associated with viral disease response in salmon were identified in five individuals of this population and drove differentiation along NMDS1, depicting the strongest physiological driver in our data. This response is characterized by strong upregulation of RSAD, IFIT5, Mx, NFX, DEXH, VAR1, X52Ro and IFI44A (Miller et al., 2017). However, the initial infectious agent panel used in this study did not indicate a viral infectious agent was present. Additional screening of these individuals with a suite of novel viruses recently identified in BC salmon (Mordecai, unpublished data) indicated that a novel strain of CTV-2 was present in some but not all of these fish. We speculate that other as yet unidentified viruses may be present in the remaining fish. This is the first data showing that the novel CTV-2 strain is present in steelhead, though other strains of this virus are known to be widely distributed throughout trout populations in the United States (Batts et al., 2011). While we do not yet know if the CTV-2 strain is pathogenic, the previously characterized strain is not considered to be highly pathogenic, with no observed mortality in rainbow trout, cutthroat trout (*Oncorhynchus clarkia*, Richardson) and kokanee salmon (*O. nerka*) following water-borne exposure to the virus (Hedrick et al., 1991). Given the individuals with CTV-2 detection show molecular evidence indicative of a viral disease state, further examination of this viral strain for pathogenic potential is warranted in steelhead.

### Temperature and host physiology

Despite cool water temperatures well below the upper temperature tolerance for *O. mykiss*, temperature had an influence on gene expression of fish upstream of Witset Falls (Yada and Tort, 2016). The most positive associations were with genes related to the complement system (C3 and C7), metabolic stress (LDHB), and oxidative stress (SEPW1). Although water temperature was not significantly correlated with gene expression in fish sampled earlier in the migration at Witset Falls, these genes were also in similar ordination space as water temperature in this group of fish. Expression of genes in the complement system indicates that a component of the innate immune system is increasing with temperature (Bowden, 2008), though many other innate immunity biomarkers had neutral or negative relationships with temperature. LDHB was also greater at warmer temperatures which is consistent with the observation that lactic acidosis in white muscle and blood generally increases with warmer temperatures in rainbow trout and steelhead (Kieffer et al., 1994; Twardek et al., 2018). Despite evidence of higher levels of metabolic products with increasing water temperature, glycolytic genes were generally not upregulated with temperature. This relationship has been observed in wild rainbow trout in similar temperature conditions as the Bulkley River, with increased resting state expression of glycolytic genes (PK and PEP3K) with temperature (Wellband and Heath, 2013). Water temperature also showed a positive association with SEPW1, which functions as an antioxidant (Whanger, 2009), and is a known temperature responsive gene (Akbarzadeh et al., 2018). SEPW1 was also elevated in adult sockeye salmon held at water temperatures >19°C compared to 13–14°C (Jeffries et al., 2014b). Water temperature was not correlated with other well-characterized thermal-responsive genes (Akbarzadeh et al., 2018) such as those encoding heat shock proteins (e.g. HSP90a and SERPINH1). Previous work evaluating the influence of thermal stress on the transcriptome of sockeye and pink salmon, *Oncorhynchus gorbuscha* (Walbaum), indicated upregulation of heat shock proteins (HSP90AA1, HSPAB1 and SERPINH1) with warm temperatures (Miller et al., 2009; Evans et al., 2011; Jeffries et al., 2014b). Our data suggest that the relatively low water temperatures observed on the Bulkley River were likely not altering protein conformation or protein aggregation in steelhead to a degree that would necessitate changes in the expression of this family of chaperone proteins.

## Conclusions

Positive detections of eight infectious agents of bacterial, viral, fungal, myxozoan and other eukaryotic origin were found in steelhead from the Bulkley River. The majority of fish sampled were infected with *Ca. B. cysticola*, *F. psychrophilum* and *S. destruens*. Despite a small sample size, interesting differences were observed across sampling sites at and above Witset Falls. Infectious loads of most identified infectious agents were greater in fish at vs. above the falls including three agents capable of causing gill disease (*L. salmonae*, *P. theridion* and *P. salmonis*). The relative load of *F. psychrophilum* was associated with the physiology of fish only at the falls, suggesting this agent may impact the migration of success of fish at this barrier. Fish upstream of the falls were further along in their migration, and observed differences in their physiology were consistent with later-stage migration. Our findings provide insight into the relationship between physiology and infectious agents and potential links to migratory outcomes in wild Steelhead. If salmonids with the highest infectious agent burdens are unable to reach spawning sites and successfully reproduce, then there may be broad scale consequences to recruitment across salmon populations. We encourage future research to evaluate the migratory culling hypothesis for long-distance salmonid migrations by combining tools in molecular genetics and biotelemetry (see Miller et al., 2011, as example). Future work should also seek to validate disease susceptibility and impacts of the agents identified in steelhead using histopathology.

## Supplementary Material

Supplemental_Tables_FINAL_coz023

## References

[ref2] Akbarzadeh A, Günther OP, Houde AL, Li S, Ming TJ, Jeffries KM, Hinch SG, Miller KM (2018) Developing specific molecular biomarkers for thermal stress in salmonids. *BMC Genomics*doi:1. 10.1186/s12864-018-5108-9 PMC6192343 30326831

[ref3] Alós J, Palmer M, Arlinghaus R (2012) Consistent selection towards low activity phenotypes when catchability depends on encounters among human predators and fish. *PLoS One* 7: e48030. doi:10.1371/journal.pone.00480300.1186/s12864-018-5108-9. 23110164 PMC3480476

[ref4] Altizer S, Bartel R, Han BA (2011) Animal migration and infectious disease risk. *Science* 331: 296–302. 21252339 10.1126/science.1194694

[ref5] Andreou D, Gozlan RE (2016) Associated disease risk from the introduced generalist pathogen *Sphaerothecum destruens*: management and policy implications. *Parasitology* 143: 1204–1210. 27216376 10.1017/S003118201600072X PMC4926270

[ref6] Andreou D, Gozlan RE, Stone D, Martin P, Bateman K, Feist SW (2011) Sphaerothecum destruenspathology in cyprinids. *Dis Aquat Organ* 95: 145–151. 10.3354/dao02356 21848122

[ref7] Arkush KD, Frasca S, Hedrick RP (1998) Pathology associated with the rosette agent, a systemic protist infecting salmonid fishes. *J Aquat Anim Health* 10: 1–11.

[ref9] Baker MR, Schindler DE (2009) Unaccounted mortality in salmon fisheries: non-retention in gillnets and effects on estimates of spawners. *J Appl Ecol* 46: 752–761. doi:10.1111/j.1365-2664.2009.01673.x.

[ref10] Barber I, Hoare D, Krause J (2000) Effects of parasites on fish behaviour: a review and evolutionary perspective. *Rev Fish Biol Fish* 10: 131–165.

[ref11] Barnes ME, Brown ML (2011) A review of *Flavobacterium psychrophilum* biology, clinical signs, and bacterial cold water disease prevention and treatment. *Open Fish Sci J* 4: 1–9.

[ref12] Bass AL, Hinch SG, Teffer AK, Patterson DA, Miller KM (2017) A survey of microparasites present in adult migrating Chinook salmon (*Oncorhynchus tshawytscha*) in south-western British Columbia determined by high-throughput quantitative polymerase chain reaction. *J Fish Dis* 40: 453–477. 28188649 10.1111/jfd.12607

[ref13] Bass AL, Hinch SG, Teffer AK, Patterson DA, Miller KM (2019) Fisheries capture and infectious agents are associated with travel rate and survival of Chinook salmon during spawning migration. *Fish Res* 209: 156–166. doi:10.1016/j.fishres.2018.09.009.

[ref14] Batts W, Yun S, Hedrick R, Winton J (2011) A novel member of the family Hepeviridae from cutthroat trout (*Oncorhynchus clarkii*). *Virus Res* 158: 116–123. 21458509 10.1016/j.virusres.2011.03.019

[ref15] Beamish RJ, Noakes DJ, McFarlane GA, Klyashtorin L, Ivanov VV, Kurashov V (1999) The regime concept and natural trends in the production of Pacific salmon. *Can J Fish Aquat Sci* 56: 516–526. doi:10.1139/f98-200.

[ref16] Bebak-Williams J, McAllister PE, Smith G, Boston R (2002) Effect of fish density and number of infectious fish on the survival of rainbow trout fry, *Oncorhynchus mykiss* (Walbaum), during epidemics of infectious pancreatic necrosis. *J Fish Dis* 25: 715–726. doi:10.1046/j.1365-2761.2002.00426.x.

[ref17] Boerlage AS, Graat EAM, Verreth JA, Jong MCM de (2011) Effect of fish size on transmission of fish-borne trematodes (*Heterophyidae*) to common carps (*Cyprinus carpio*) and implications for intervention. *Aquaculture* 321: 179–184.

[ref18] Bowden TJ (2008) Modulation of the immune system of fish by their environment. *Fish Shellfish Immunol* 25: 373–383. 18562213 10.1016/j.fsi.2008.03.017

[ref19] Bowerman T, Roumasset A, Keefer ML, Sharpe CS, Caudill CC (2018) Prespawn mortality of female Chinook salmon increases with water temperature and percent hatchery origin. *Trans Am Fish Soc* 147: 31–42.

[ref20] Bradford MJ, Lovy J, Patterson DA, Speare DJ, Bennett WR, Stobbart AR, Tovey CP (2010) Parvicapsula minibicornis infections in gill and kidney and the premature mortality of adult sockeye salmon (*Oncorhynchus nerka*) from Cultus Lake, British Columbia. *Can J Fish Aquat Sci* 67: 673–683.

[ref22] Brett JR (1971) Energetic responses of salmon to temperature. A study of some thermal relations in the physiology and freshwater ecology of sockeye salmon (*Oncorhynchus nerka*). *Integr Comp Biol* 11: 99–113.

[ref24] Casadevall A, Pirofski LA (1999) Host–pathogen interactions: redefining the basic concepts of virulence and pathogenicity. *Infect Immun* 67: 3703–3713. 10417127 10.1128/iai.67.8.3703-3713.1999 PMC96643

[ref25] Castro R, Jouneau L, Tacchi L, Macqueen DJ, Alzaid A, Secombes CJ, Martin SAM, Boudinot P (2015) Disparate developmental patterns of immune responses to bacterial and viral infections in fish. *Sci Rep* 5: 15458. 26487553 10.1038/srep15458 PMC4614352

[ref26] Cook KV (2018) Molecular, physiological and behavioural responses to capture in pacific salmon commercial fisheries: implications for postrelease survival of non-target salmon species. PhD Thesis, University of British Columbia, Vancouver, Canada, pp 265.

[ref27] Cooke SJ, Crossin GT, Hinch SG (2011) Pacific salmon migration: completing the cycle In Farrell AP, ed, Encyclopedia of Fish Physiology: From Genome to Environment 3. Academic Press, San Diego, pp. 1945–1952.

[ref28] Cooke SJ, Crossin GT, Patterson DA, English KK, Hinch SG, Young JL, Alexander RF, Healey MC, Van Der Kraak G, Farrell AP (2005) Coupling non-invasive physiological assessments with telemetry to understand inter-individual variation in behaviour and survivorship of sockeye salmon: development and validation of a technique. *J Fish Biol* 67: 1342–1358. doi:10.1111/j.1095-8649.2005.00830.x.

[ref29] Cooke SJ, Hinch SG, Crossin GT, Patterson DA, English KK, Healey MC, Shrimpton JM, Van Der Kraak G, Farrell AP (2006) Mechanistic basis of individual mortality in pacific salmon during spawning migrations. *Ecology* 87:1575–1586. doi:10.1890/0012-9658(2006)87[1575:MBOIMI]2.0.CO;2. 16869433

[ref30] Cornwell ER, Bellmund CA, Groocock GH, Wong PT, Hambury KL, Getchell RG, Bowser PR (2013) Fin and gill biopsies are effective nonlethal samples for detection of viral hemorrhagic septicemia virus genotype IVb. *J Vet Diagnostic Invest* 25: 203–209. doi:10.1177/1040638713476865. 23404480

[ref31] Cotner JB, Biddanda BA (2002) Small players, large role: microbial influence on biogeochemical processes in pelagic aquatic ecosystems. *Ecosystems* 5: 105–121.

[ref34] Currie JLM, Woo PTK (2007) Susceptibility of sexually mature rainbow trout, *Oncorhynchus mykiss* to experimental cryptobiosis caused by *Cryptobia salmositica* *Parasitol Res* 101: 1057–1067 doi:10.1007/s00436-007-0586-8. 17582534

[ref36] Diaz Pauli B, Wiech M, Heino M, Utne-Palm AC (2015) Opposite selection on behavioural types by active and passive fishing gears in a simulated guppy *Poecilia reticulata* fishery. *J Fish Biol* 86: 1030–1045. doi:10.1111/jfb.12620. 25619538

[ref39] Evans TG, Hammill E, Kaukinen K, Schulze AD, Patterson DA, English KK, Curtis JMR, Miller KM (2011) Transcriptomics of environmental acclimatization and survival in wild adult Pacific sockeye salmon (*Oncorhynchus nerka*) during spawning migration. *Mol Ecol* 20: 4472–4489. 21951593 10.1111/j.1365-294X.2011.05276.x

[ref42] Fry FEJ (1971) The effect of environmental factors on the physiology of fish In Hoar WS, Randall DJ, eds, Fish Physiology Vol 6 Academic Press, New York, USA, pp. 1–99.

[ref43] Gibson-Reinemer DK, Chick JH, VanMiddlesworth TD, VanMiddlesworth M, Casper AF (2017) Widespread and enduring demographic collapse of invasive common carp (*Cyprinus carpio*) in the upper Mississippi River system. *Biol Invasions* 19: 1905–1916. doi:10.1007/s10530-017-1405-5.

[ref46] Hedrick RP, Yun WH, Wingfield A (1991) Small RNA virus isolated from salmonid fishes in California, USA. *Can J Fish Aquat Sci* 48: 99–104.

[ref48] Hessenauer JM, Vokoun JC, Suski CD, Davis J, Jacobs R, O’Donnell E (2015) Differences in the metabolic rates of exploited and unexploited fish populations: a signature of recreational fisheries induced evolution? *PLoS One* 10: e012833. 10.1371/journal.pone.0128336 PMC4454643 26039091

[ref49] Hinch SG, Bratty J (2000) Effects of swim speed and activity pattern on success of adult sockeye salmon migration through an area of difficult passage. *Trans Am Fish Soc* 129: 598–606. doi: 10.1577/1548-8659(2000)129<0598:eossaa>2.0.co;2.

[ref50] Hinch SG, Cooke SJ, Farrell AP, Miller KM, Lapointe M, Patterson DA (2012) Dead fish swimming: a review of research on the early migration and high premature mortality in adult Fraser River sockeye salmon *Oncorhynchus nerka*. *J Fish Biol* 81: 576–599. 22803725 10.1111/j.1095-8649.2012.03360.x

[ref52] Hollins J, Thambithurai D, Koeck B, Crespel A, Bailey DM, Cooke SJ, Lindström J, Parsons KJ, Killen SS (2018) A physiological perspective on fisheries-induced evolution. *Evol Appl* 11: 561–576. 29875803 10.1111/eva.12597 PMC5978952

[ref53] Holt RA (1987) Cytophaga psychrophila, the causative agent of bacterial cold-water disease in salmonid fish. PhD thesis. Oregon State University, Corvallis, OR, pp 182.

[ref55] Jeffries KM et al. (2014a) Immune response genes and pathogen presence predict migration survival in wild salmon smolts. *Mol Ecol* 23: 5803–5815. 25354752 10.1111/mec.12980

[ref57] Jeffries KM, Hinch SG, Sierocinski T, Clark TD, Eliason EJ, Donaldson MR, Li S, Pavlidis P, Miller KM (2012a) Consequences of high temperatures and premature mortality on the transcriptome and blood physiology of wild adult sockeye salmon (*Oncorhynchus nerka*). *Ecol Evol* 2: 1747–1764. 22957178 10.1002/ece3.274 PMC3434914

[ref58] Jeffries KM, Hinch SG, Sierocinski T, Pavlidis P, Miller KM (2014b) Transcriptomic responses to high water temperature in two species of Pacific salmon. *Evol Appl* 7: 286–300. 24567748 10.1111/eva.12119 PMC3927889

[ref60] Keefer ML, Peery CA, High B (2009) Behavioral thermoregulation and associated mortality trade-offs in migrating adult steelhead (*Oncorhynchus mykiss*): variability among sympatric populations. *Can J Fish Aquat Sci* 66: 1734–1747. doi:10.1139/F09-131.

[ref61] Kent L, Groff JM, Morrison JK, Yasutake WT, Holt RA (1989) Spiral swimming behavior due to cranial and vertebral lesions associated with *Cytophaga psychrophila* infections in salmonid fishes. *Dis Aquat Organ* 6: 11–16. doi:10.3354/dao006011.

[ref62] Kent M (2011) Infectious diseases and potential impacts on survival of Fraser River sockeye salmon. Cohen Commission Technical Report 1, pp 58.

[ref63] Kieffer JD, Currie S, Tufts BL (1994) Effects of environmental temperature on the metabolic and acid–base responses of rainbow trout to exhaustive exercise. *J Exp Biol* 194: 299–317. 9317846 10.1242/jeb.194.1.299

[ref64] Kruskal JB (1964) Multidimensional scaling by optimizing goodness of fit to a nonmetric hypothesis. *Psychometrika* 29: 1–27. doi:10.1007/BF02289565.

[ref65] Kubokawa K, Watanabe T, Yoshioka M, Iwata M (1999) Effects of acute stress on plasma cortisol, sex steroid hormone, and glucose levels in male and female sockeye salmon during the breeding season. *Aquaculture* 172: 335–349.

[ref66] Lawson Handley L (2015) How will the ‘molecular revolution’ contribute to biological recording? *Biol J Linn Soc* 115: 750–766.

[ref68] Livak KJ, Schmittgen TD (2001) Analysis of relative gene expression data using real-time quantitative PCR and the 2∆∆C(T) method. *Methods* 25: 402–408. 11846609 10.1006/meth.2001.1262

[ref70] Louison MJ, Adhikari S, Stein JA, Suski CD (2017) Hormonal responsiveness to stress is negatively associated with vulnerability to angling capture in fish. *J Exp Biol* 220: 2529–2535. 28724703 10.1242/jeb.150730

[ref73] Marcos-López M, Gale P, Oidtmann BC, Peeler EJ (2010) Assessing the impact of climate change on disease emergence in freshwater fish in the United Kingdom. *Transbound Emerg Dis* 57: 293–304. doi:10.1111/j.1865-1682.2010.01150.x. 20561287

[ref74] Marty G, Quinn T II, Carpenter G, Meyers TR, Willits N (2003) Role of disease in abundance of a Pacific herring (*Clupea pallasi*) population. *Can J Fish Aquat Sci* 1265: 1258–1265.

[ref75] Maule AG, Tripp RA, Kaattari SL, Schreck CB (1989) Stress alters immune function and disease resistance in Chinook salmon (*Oncorhynchus tshawytscha*). *J Endocrinol* 120: 135–142. doi:10.1677/joe.0.1200135. 2918264

[ref77] Mendonca HL, Arkush KD (2004) Development of PCR-based methods for detection of *Sphaerothecum destruens* in fish tissues. *Dis Aquat Organ* 61: 187–197. doi:10.3354/dao061187. 15609874

[ref78] Miller KM, Gardner IA, Vanderstichel R, Burnley T, Angela D, Li S, Tabata A, Kaukinen KH, Ming TJ, Ginther NG (2016) Report on the performance evaluation of the Fluidigm BioMark platform for high-throughput microbe monitoring in salmon. DFO Canadian Science Advisory Secretariat Document 2016/038, p 282.

[ref79] Miller KM, Günther OP, Li S, Kaukinen KH, Ming TJ (2017) Molecular indices of viral disease development in wild migrating salmon. *Conserv Physiol* 5: doi:10.1093/conphys/cox036. PMC5499884 28702195

[ref80] Miller KM et al. (2011) Genomic signatures predict migration and spawning failure in wild Canadian salmon. *Science* 331: 214–217. 21233388 10.1126/science.1196901

[ref81] Miller KM, Schulze AD, Ginther N, Li S, Patterson DA, Farrell AP, Hinch SG (2009) Salmon spawning migration: metabolic shifts and environmental triggers. *Comp Biochem Physiol Part D Genomics Proteomics* 4: 75–89. doi:10.1016/j.cbd.2008.11.002. 20403740

[ref82] Miller KM, Teffer A, Tucker S, Shaorong L, Schulze AD, Trudel M, Juanes F, Tabata A, Haukinen KH, Ginther NG et al. (2014) Infectious disease, shifting climates, and opportunistic predators: cumulative factors potentially impacting wild salmon declines. *Evol Appl* 7: 812–855. doi:10.1111/eva.12164. 25469162 PMC4227861

[ref83] Mitchell SO, Steinum TM, Toenshoff ER, Kvellestad A, Falk K, Horn M, Colquhoun DJ (2013) Candidatus Branchiomonas cysticolais a common agent of epitheliocysts in seawater-farmed Atlantic salmon *Salmo salar* in Norway and Ireland. *Dis Aquat Organ* 103: 35–43. doi:10.3354/dao02563. 23482383

[ref84] Molina-Fernández D, Malagón D, Gómez-Mateos M, Benítez R, Martín-Sánchez J, Adroher FJ (2015) Fishing area and fish size as risk factors of *Anisakis* infection in sardines (*Sardina pilchardus*) from Iberian waters, southwestern Europe. *Int J Food Microbiol* 203: 27–34. doi:10.1016/j.ijfoodmicro.2015.02.024. 25770431

[NRC] National Research Council (1996) Upstream: Salmon and Society in the Pacific Northwest, Society. The National Academies Press, Washington, DC, p. 472.

[ref87] Nematollahi A, Decostere A, Pasmans F, Haesebrouck F (2003) Flavobacterium psychrophiluminfections in salmonid fish. *J Fish Dis* 26: 563–574. 10.1046/j.1365-2761.2003.00488.x 14653314

[ref88] Nowak BF, LaPatra SE (2006) Epitheliocystis in fish. *J Fish Dis* 29: 573–588. 17026667 10.1111/j.1365-2761.2006.00747.x

[ref89] Nylund S, Andersen L, Sævareid I, Plarre H, Watanabe K, Arnesen CE, Karlsbakk E, Nylund A (2011) Diseases of farmed Atlantic salmon Salmo salar associated with infections by the microsporidian *Paranucleospora theridion*. Dis Aquat Organ 94: 41–57. 21553567 10.3354/dao02313

[ref90] Nylund S, Nylund A, Watanabe K, Arnesen CE, Karlsbakk E (2010) Paranucleospora theridionn. gen., n. sp. (*Microsporidia*, Enterocytozoonidae) with a life cycle in the salmon louse (*Lepeophtheirus salmonis*, *Copepoda*) and Atlantic salmon (*Salmo salar*). *J Eukaryot Microbiol* 57: 95–114. 10.1111/j.1550-7408.2009.00451.x 20070452

[ref91] Okland F, Erkinaro J, Moen K, Niemelä E, Fiske P, McKinley RS, Thorstad EB (2001) Return migration of Atlantic salmon in the river Tana: phases of migratory behaviour. *J Fish Biol* 59: 862–874. doi:10.1006/jfbi.2001.1701.

[ref92] Pearson WH, Elston RA, Bienert RW, Drum AS, Antrim LD (1999) Why did the Prince William Sound, Alaska, Pacific herring (*Clupea pallasi*) fisheries collapse in 1993 and 1994? Review of hypotheses. *Can J Fish Aquat Sci* 56: 711–737. doi:10.1139/f98-207.

[ref93] Peeler EJ, Taylor NG (2011) The application of epidemiology in aquatic animal health—opportunities and challenges. *Vet Res* 42: 94. 21834990 10.1186/1297-9716-42-94 PMC3182899

[ref95] Philipp DP, Claussen JE, Koppelman JB, Stein JA, Cooke SJ, Suski CD, Wahl DH, Sutter DAH, Arlinghaus R (2015) Fisheries-induced evolution in largemouth bass: linking vulnerability to angling, parental care, and fitness. *Am Fish Soc Symp* 82: 223–234.

[ref96] Pickering AD, Pottinger TG (1989) Stress responses and disease resistance in salmonid fish: effects of chronic elevation of plasma cortisol. *Fish Physiol Biochem* 7: 253–258. doi:10.1007/BF00004714. 24221779

[ref98] Price PW (1980) Evolutionary Biology of Parasites. Princeton University Press, Princeton, New Jersey, p. 237.

[ref100] R Core Team (2015) R: a language and environment for statistical computing. R Foundation for Statistical Computing, Vienna, Austria, https://www.R-project.org/.

[ref101] Richards EL, Oosterhout C van, Cable J (2010) Sex-specific differences in shoaling affect parasite transmission in guppies. *PLoS One* 5: e13285. 20949014 10.1371/journal.pone.0013285 PMC2952601

[ref102] Risely A, Klaassen M, Hoye BJ (2018) Migratory animals feel the cost of getting sick: a meta-analysis across species. *J Anim Ecol* 87: 301–314. doi:10.1111/1365-2656.12766. 28994103

[ref103] Rohlenová K, Morand S, Hyrl P, Tolarová S, Flajhans M, Imková A (2011) Are fish immune systems really affected by parasites? An immunoecological study of common carp (*Cyprinus carpio*). *Parasit Vectors* 4: 120. 21708010 10.1186/1756-3305-4-120 PMC3155493

[ref104] Rottmann RW, Francis-Floyd R, Durborow R (1992) The role of stress in fish disease. Southern Regional Aquaculture Center p 4. doi:10.1046/j.1365-2761.2001.00314.x.

[ref105] Sandblom E, Clark TD, Hinch SG, Farrell AP (2009) Sex-specific differences in cardiac control and hematology of sockeye salmon (*Oncorhynchus nerka*) approaching their spawning grounds. *Am J Physiol Integr Comp Physiol* 297: 1136–1143. doi:10.1152/ajpregu.00363.2009. 19675278

[ref106] Schachte JH (1983) Bacterial gill disease In Carey TG, Meyer FP, Warren JW, eds, Guide to Integrated Fish Health Management in the Great Lakes Basin. Ann Arbor, MI, USA, Great Lakes Fishery Commission, pp. 262.

[ref107] Schaffer WM (2004) Life histories, evolution, and salmonids In Stearns S, Hendry A, eds, Evolution Illuminated: Salmon and Their Relatives. Oxford University Press, UK, pp. 20–61.

[ref108] Schmidt-Posthaus H, Polkinghorne A, Nufer L, Schifferli A, Zimmermann DR, Segner H, Steiner P, Vaughan L (2012) A natural freshwater origin for two chlamydial species, Candidatus Piscichlamydia salmonis and Candidatus Clavochlamydia salmonicola, causing mixed infections in wild brown trout (*Salmo trutta*). *Environ Microbiol* 14: 2048–2057. doi:10.1111/j.1462-2920.2011.02670.x. 22176683

[ref109] Scott WB, Crossman EJ (1973) Freshwater fishes of Canada. Bulletin 184. Fisheries Research Board of Canada 1973, pp 966.

[ref110] Sepahi A, Heidarieh M, Mirvaghefi A, Rafiee GR, Farid M, Sheikhzadeh N (2013) Effects of water temperature on the susceptibility of rainbow trout to streptococcus agalactiae. *Acta Sci Vet* 41: 1–5.

[ref112] Smith VH (2007) Microbial diversity–productivity relationships in aquatic ecosystems. *FEMS Microbiol Ecol* 62: 181–186. 17868363 10.1111/j.1574-6941.2007.00381.x

[ref113] Speare DJ, Lovy J (2012) Loma salmonae and related species In PTK W, Buchman K, eds, Fish parasites: Pathobiology and Protection, CABI, London, UK, pp. 109–130.

[ref114] Starliper CE (2011) Bacterial coldwater disease of fishes caused by *Flavobacterium psychrophilum*. *J Adv Res* 2: 97–108. doi:10.1016/j.jare.2010.04.001.

[ref115] Steen RP, Quinn TP (1999) Egg burial depth by sockeye salmon (*Oncorhynchus nerka*): implications for survival of embryos and natural selection on female body size. *Can J Zool* 77: 836–841.

[ref116] Steinbach Elwell LC, Stromberg KE, Ryce EKN, Bartholomew J (2009) Whirling disease in the United States: a summary of progress in research and management 2009. Technical report, p 66.

[ref118] Suttle CA (2005) Viruses in the sea. *Nature* 437: 356–361. 16163346 10.1038/nature04160

[ref119] Taguchi YH, Oono Y (2005) Relational patterns of gene expression via non-metric multidimensional scaling analysis. *Bioinformatics* 21: 730–740. doi:10.1093/bioinformatics/bti067. 15509613

[ref120] Tavares GC, Costa FA, RRD Santos, Barony GM, CAG Leal, HCP Figueiredo (2016) Nonlethal sampling methods for diagnosis of *Streptococcus agalactiae* infection in Nile tilapia, *Oreochromis niloticus* (L.). *Aquaculture* 454:237–242. doi:10.1016/j.aquaculture.2015.12.028.

[ref122] Teffer AK, Hinch SG, Miller KM, Patterson DA, Farrell AP, Cooke SJ, Bass AL, Szekeres P, Juanes F (2017) Capture severity, infectious disease processes and sex influence post-release mortality of sockeye salmon bycatch. *Conserv Physiol* 5: doi:10.1093/conphys/cox017. PMC5569998 28852514

[ref123] Thoen E, Evensen Ø, Skaar I (2016) Factors influencing *Saprolegnia* spp. spore numbers in Norwegian salmon hatcheries. *J Fish Dis* 39: 657–665. doi:10.1111/jfd.12392. 26123005

[ref124] Toenshoff ER, Kvellestad A, Mitchell SO, Steinum T, Falk K, Colquhoun DJ, Horn M (2012) A novel betaproteobacterial agent of gill epitheliocystis in seawater farmed Atlantic salmon (*Salmo salar*). *PLoS One* 7: e32696. doi:10.1371/journal.pone.0032696. 22427865 PMC3299688

[ref125] Traxler GS, Richard J, McDonald TE (1998) Ichthyophthirius multifiliis (Ich) epizootics in spawning sockeye salmon in British Columbia. *Can J Aquat Anim Health* 10: 143–151. doi:10.1577/1548-8667(1998)010<0143:IMIEIS>2.0.CO;2.

[ref126] Traxler GS, Roome JR, Lauda KA, LaPatra S (1997) Appearance of infectious hematopoietic necrosis virus (IHNV) and neutralizing antibodies in sockeye salmon *Onchorynchus nerka* during their migration and maturation period. *Dis Aquat Organ* 28: 31–38. doi:10.3354/dao028031.

[ref127] Tucker S, Li S, Kaukinen KH, Patterson DA, Miller KM (2018) Distinct seasonal infectious agent profiles in life-history variants of juvenile Fraser River Chinook salmon: an application of high-throughput genomic screening. *PLoS One* 13: e0195472. 29672620 10.1371/journal.pone.0195472 PMC5908190

[ref128] Twardek WM, Elmer LK, Beere MC, Cooke SJ, Danylchuk AJ (2019) Consequences of fishery gear type and handling practices on capture and release of wild steelhead on the Bulkley River. *N Am J Fish Manag* 39: 254–269.

[ref129] Twardek WM, Gagne TO, Elmer LK, Cooke SJ, Beere MC, Danylchuk AJ (2018) Consequences of catch-and-release angling on the physiology, behaviour and survival of wild steelhead *Oncorhynchus mykiss* in the Bulkley River, British Columbia. *Fish Res* 206: 235–246.

[ref130] Uribe C, Folch H, Enriquez R, Moran G (2011) Innate and adaptive immunity in teleost fish: a review. *Vet Med* 56: 486–503.

[ref131] Wagner GN, McKinley RS, Bjørn PA, Finstad B (2003) Physiological impact of sea lice on swimming performance of Atlantic salmon. *J Fish Biol* 62: 1000–1009. doi:10.1046/j.1095-8649.2003.00091.x.

[ref133] Wedemeyer G (1970) The role of stress in the disease resistance of fishes. In Snieszko S., ed, Symposium on Diseases of Fishes and Shellfishes, Vol 5 American Fisheries Society, Washington, D.C., U.S.A, pp 30–35.

[ref134] Welch DW, Jacobs MJ, Lydersen H, Porter AD, Williams S, Muirhead Y (2009) Acoustic telemetry measurements of survival and movements of adult steelhead (*Oncorhynchus mykiss*) within the Skeena and Bulkley Rivers, 2008. Kintama Research Corporation, Final Report to the B.C. Ministry of the Environment, 50 pages.

[ref135] Wellband KW, Heath DD (2013) Environmental associations with gene transcription in Babine Lake rainbow trout: evidence for local adaptation. *Ecol Evol* 3: 1194–1208. doi:10.1002/ece3.531. 23762507 PMC3678475

[ref136] Whanger PD (2009) Selenoprotein expression and function—selenoprotein W. *Biochim Biophys Acta* 1790: 1448–1452. 19464347 10.1016/j.bbagen.2009.05.010

[ref137] Wiik-Nielsen J, Gjessing M, Solheim HT, Litlabø A, Gjevre AG, Kristoffersen AB, Powell MD, Colquhoun DJ (2017) Ca. Branchiomonas cysticola, Ca. Piscichlamydia salmonisand salmon gill pox virus transmit horizontally in Atlantic salmon held in fresh water. *J Fish Dis* 40: 1387–1394. 10.1111/jfd.12613 28261804

[ref138] Yada T, Tort L (2016) Stress and disease resistance: immune system and immunoendocrine interactions In Schreck CB, Tort L, Farrell AP, Brauner CJ, eds, Biology of Stress in Fish, Fish Physiology Vol 35 Academic Press, New York, USA, pp 365–403.

[ref139] Zuk M, Stoehr AM (2002) Immune Defense and Host Life History. *Am Nat* 160: S9–S22. doi: 10.2307/3079266. 18707455

