# Supplementary material for: Evidence of a hydraulically challenging reach serving as a barrier for the upstream migration of infection-burdened adult steelhead
Source: Conserv Physiol. 2019 Jun 6;7(1):coz023. doi: 10.1093/conphys/coz023 (PMC6553125; doi:10.1093/conphys/coz023)
Supplement: Supplemental_Tables_FINAL_coz023 [file supplemental_tables_final_coz023.docx]

Supplementary Table 1. The prevalence of 47 viruses, as well as bacterial, fungal, and protist parasites in adult Bulkley River steelhead. All but *Kudoa thyrsites* are suspected to cause disease in salmon. For each assay the abbreviation, infectious agent name, type of infectious agent, prevalence, primer/probe sequences, and efficiency are presented. Assays were first run against a pooled sample, and only positive detections were included in the final run. Therefore, assays that have a prevalence of zero depict the assay efficiencies from the pooled sample run.

| **Assay name** | **Infectious agent name** | **Type** | **Prevalence (%)** | **Primer and probe sequences** | **Efficiency** |
| --- | --- | --- | --- | --- | --- |
|  |  |  |  |  |  |
| ae_hyd | *Aeromonas* | Bacterium | 0 | F—ACCGCTGCTCATTACTCTGATG | 1.21 |
|  | *hydrophila* |  |  | R—CCAACCCAGACGGGAAGAA |  |
|  |  |  |  | P—TGATGGTGAGCTGGTTG |  |
| ae_sal | *Aeromonas* | Bacterium | 0 | F—TAAAGCACTGTCTGTTACC | 1.14 |
|  | salmonicida |  |  | R—GCTACTTCACCCTGATTGG |  |
|  |  |  |  | P—ACATCAGCAGGCTTCAGAGTCACTG |  |
| re_sal | *Renibacterium* | Bacterium | 0 | F—CAACAGGGTGGTTATTCTGCTTTC | 1.26 |
|  | salmoninarum |  |  | R—CTATAAGAGCCACCAGCTGCAA |  |
|  |  |  |  | P—CTCCAGCGCCGCAGGAGGAC |  |
| c_b_cys | *Candidatus* | Bacterium | 80 | F—AATACATCGGAACGTGTCTAGTG | 0.91 |
|  | Branchiomonas |  |  | R—GCCATCAGCCGCTCATGTG |  |
|  | cysticola |  |  | P—CTCGGTCCCAGGCTTTCCTCTCCCA |  |
| ye_ruc | *Yersinia* | Bacterium | 0 | F—TGCCGCGTGTGTGAAGAA | 1.26 |
|  | ruckeri |  |  | R—ACGGAGTTAGCCGGTGCTT |  |
|  |  |  |  | P—AATAGCACTGAACATTGAC |  |
| fl_psy | *Flavobacterium* | Bacterium | 92.5 | F—GATCCTTATTCTCACAGTACCGTCAA | 0.82 |
|  | *psychrophilum* |  |  | R—TGTAAACTGCTTTTGCACAGGAA |  |
|  |  |  |  | P—AAACACTCGGTCGTGACC |  |
| mo_vis | *Moritella* | Bacterium | 0 | F—CGTTGCGAATGCAGAGGT | 1.23 |
|  | viscosa |  |  | R—AGGCATTGCTTGCTGGTTA |  |
|  |  |  |  | P—TGCAGGCAAGCCAACTTCGACA |  |
| pch_sal | *Piscichlamydia* | Bacterium | 7.5 | F—TCACCCCCAGGCTGCTT | 1.08 |
|  | salmonis |  |  | R—GAATTCCATTTCCCCCTCTTG |  |
|  |  |  |  | P—CAAAACTGCTAGACTAGAGT |  |
| pisck_sal | *Piscirickettsia* | Bacterium | 0 | F—TCTGGGAAGTGTGGCGATAGA | 1.18 |
|  | salmonis |  |  | R—TCCCGACCTACTCTTGTTTCATC |  |
|  |  |  |  | P—TGATAGCCCCGTACACGAAACGGCATA |  |
| rlo | *Rickettsia-like* | Bacterium | 0 | F—GGCTCAACCCAAGAACTGCTT | 1.14 |
|  | organism |  |  | R—GTGCAACAGCGTCAGTGACT |  |
|  |  |  |  | P—CCCAGATAACCGCCTTCGCCTCCG |  |
| sch | *Gill* | Bacterium | 0 | F—GGGTAGCCCGATATCTTCAAAGT | 1.29 |
|  | chlamydia |  |  | R—CCCATGAGCCGCTCTCTCT |  |
|  |  |  |  | P—TCCTTCGGGACCTTAC |  |
| te_mar | *Tenacibaculum* | Bacterium | 0 | F—TGCCTTCTACAGAGGGATAGCC | 1.22 |
|  | maritimum |  |  | R—CTATCGTTGCCATGGTAAGCCG |  |
|  |  |  |  | P—CACTTTGGAATGGCATCG |  |
| vi_ang | *Vibrio* | Bacterium | 0 | F—CCGTCATGCTATCTAGAGATGTATTTGA | 1.23 |
|  | anguillarum |  |  | R—CCATACGCAGCCAAAAATCA |  |
|  |  |  |  | P—TCATTTCGACGAGCGTCTTGTTCAGC |  |
| vi_sal | *Vibrio* | Bacterium | 0 | F—GTGTGATGACCGTTCCATATTT | 1.16 |
|  | salmonicida |  |  | R—GCTATTGTCATCACTCTGTTTCTT |  |
|  |  |  |  | P—TCGCTTCATGTTGTGTAATTAGGAGCGA |  |
| pmcv | Piscine | Virus | 0 | F—TTCCAAACAATTCGAGAAGCG | 1.25 |
|  | totivirus (CMS) |  |  | R—ACCTGCCATTTTCCCCTCTT |  |
|  |  |  |  | P—CCGGGTAAAGTATTTGCGTC |  |
| ver | Viral encephalopathy | Virus | 0 | F—TTCCAGCGATACGCTGTTGA | 1.32 |
|  | and retinopathy virus |  |  | R—CACCGCCCGTGTTTGC |  |
|  |  |  |  | P—AAATTCAGCCAATGTGCCCC |  |
| vhsv | Viral haemorrhagic | Virus | 0 | F—ATGAGGCAGGTGTCGGAGG | 1.15 |
|  | septicaemia |  |  | R—TGTAGTAGGACTCTCCCAGCATCC |  |
|  | virus |  |  | P—TACGCCATCATGATGAGT |  |
| omv | Salmonid | Virus | 0 | F—GCCTGGACCACAATCTCAATG | 1.21 |
|  | herpesvirus |  |  | R—CGAGACAGTGTGGCAAGACAAC |  |
|  |  |  |  | P—CCAACAGGATGGTCATTA |  |
| sav | Salmon | Virus | 0 | F—CCGGCCCTGAACCAGTT | 1.14 |
|  | alphavirus |  |  | R—GTAGCCAAGTGGGAGAAAGCT |  |
|  |  |  |  | P—TCGAAGTGGTGGCCAG |  |
| ven | Viral | Virus | 0 | F—CGTAGGGCCCCAATAGTTTCT | 1.27 |
|  | erythrocytic |  |  | R—GGAGGAAATGCAGACAAGATTTG |  |
|  | necrosis virus |  |  | P—TCTTGCCGTTATTTCCAGCACCCG |  |
| pspv | Pacific salmon | Virus | 0 | F—CCCTCAGGCTCCGATTTTTAT | 1.21 |
|  | parvovirus |  |  | R—CGAAGACAACATGGAGGTGACA |  |
|  |  |  |  | P—CAATTGGAGGCAACTGTA |  |
| prv | Piscine reovirus | Virus | 0 | F—TGCTAACACTCCAGGAGTCATTG | 1.2 |
|  | (HSMI, CMS) |  |  | R—TGAATCCGCTGCAGATGAGTA |  |
|  |  |  |  | P—CGCCGGTAGCTCT |  |
| ihnv | Infectious | Virus | 0 | F—AGAGCCAAGGCACTGTGCG | 1.14 |
|  | haematopoietic |  |  | R—TTCTTTGCGGCTTGGTTGA |  |
|  | necrosis virus |  |  | P—TGAGACTGAGCGGGACA |  |
| ipnv | Infectious | Virus | 0 | F—GCCAAGATGACCCAGTCCAT | 1.02 |
|  | pancreatic |  |  | R—TGACAGCTTGACCCTGGTGAT |  |
|  | necrosis virus |  |  | P—CCGACCGAGAACAT |  |
| sav | Salmon alphavirus | Virus | 0 | F—CCGCACGGTTGTAAGATCAGT | 1.25 |
|  |  |  |  | R—CGTCCGGAATGTTGATGGA |  |
|  |  |  |  | P—CTCCTGGCCCTCGAT |  |
| cr_sal | *Cryptobia* | Parasite | 0 | F—TCAGTGCCTTTCAGGACATC | 1.16 |
|  | salmositica |  |  | R—GAGGCATCCACTCCAATAGAC |  |
|  |  |  |  | P—AGGAGGACATGGCAGCCTTTGTAT |  |
| ce_sha | *Ceratonova* | Parasite | 0 | F—CCAGCTTGAGATTAGCTCGGTAA | 1.33 |
|  | *shasta* |  |  | R—CCCCGGAACCCGAAAG |  |
|  |  |  |  | P—CGAGCCAAGTTGGTCTCTCCGTGAAAAC |  |
| de_sal | *Dermocystidium* | Parasite | 0 | F—CAGCCAATCCTTTCGCTTCT | 1.19 |
|  | salmonis |  |  | R—GACGGACGCACACCACAGT |  |
|  |  |  |  | P—AAGCGGCGTGTGCC |  |
| fa_mar | *Facilispora* | Parasite | 0 | F—AGGAAGGAGCACGCAAGAAC | 1.32 |
|  | margolisi |  |  | R—CGCGTGCAGCCCAGTAC |  |
|  |  |  |  | P—TCAGTGATGCCCTCAGA |  |
| gy_sal | *Gyrodactylus* | Parasite | 0 | F—CGATCGTCACTCGGAATCG | 1.18 |
|  | salaris |  |  | R—GGTGGCGCACCTATTCTACA |  |
|  |  |  |  | P—TCTTATTAACCAGTTCTGC |  |
| ic_mul | *Ichthyophthirius multifiliis* | Parasite | 0 | F—AAATGGGCATACGTTTGCAAA | 1.01 |
|  | multifiliis |  |  | R—AACCTGCCTGAAACACTCTAATTTTT |  |
|  |  |  |  | P—ACTCGGCCTTCACTGGTTCGACTTGG |  |
| ku_thy | *Kudoa thyrsites* | Parasite | 0 | F—TGGCGGCCAAATCTAGGTT | 1.09 |
|  |  |  |  | R—GACCGCACACAAGAAGTTAATCC |  |
|  |  |  |  | P—TATCGCGAGAGCCGC |  |
| lo_sal | *Loma salmonae* | Parasite | 10 | F—GGAGTCGCAGCGAAGATAGC | 0.93 |
|  |  |  |  | R—CTTTTCCTCCCTTTACTCATATGCTT |  |
|  |  |  |  | P—TGCCTGAAATCACGAGAGTGAGACTACCC |  |
| my_arc | *Myxobolus* | Parasite | 0 | F—TGGTAGATACTGAATATCCGGGTTT | 1.14 |
|  | arcticus |  |  | R—AACTGCGCGGTCAAAGTTG |  |
|  |  |  |  | P—CGTTGATTGTGAGGTTGG |  |
| my_ins | *Myxobolus* | Parasite | 0 | F—CCAATTTGGGAGCGTCAAA | 1.2 |
|  | insidiosus |  |  | R—CGATCGGCAAAGTTATCTAGATTCA |  |
|  |  |  |  | P—CTCTCAAGGCATTTAT |  |
| my_cer | *Myxobolus* | Parasite | 0 | F—GCCATTGAATTTGACTTTGGATTA | 0.95 |
|  | cerebralis |  |  | R—ACCATTCATGTAAGCCCGAACT |  |
|  |  |  |  | P—TCGAAGCCTTGACCATCTTTTGGCC |  |
| ne_per | *Neoparamoeba* | Parasite | 0 | F—GTTCTTTCGGGAGCTGGGAG | 1.29 |
|  | perurans |  |  | R—GAACTATCGCCGGCACAAAAG |  |
|  |  |  |  | P—CAATGCCATTCTTTTCGGA |  |
| nu_sal | *Nucleospora* | Parasite | 0 | F—GCCGCAGATCATTACTAAAAACCT | 1.22 |
|  | salmonis |  |  | R—CGATCGCCGCATCTAAACA |  |
|  |  |  |  | P—CCCCGCGCATCCAGAAATACGC |  |
| pa_ther | *Para-* | Parasite | 5 | F—CGGACAGGGAGCATGGTATAG | 0.94 |
|  | nucleospora |  |  | R—GGTCCAGGTTGGGTCTTGAG |  |
|  | *theridion* |  |  | P—TTGGCGAAGAATGAAA |  |
| pa_pse | *Parvicapsula* | Parasite | 0 | F—CAGCTCCAGTAGTGTATTTCA | 1.13 |
|  | *pseudo-* |  |  | R—TTGAGCACTCTGCTTTATTCAA |  |
|  | *branchicola* |  |  | P—CGTATTGCTGTCTTTGACATGCAGT |  |
| pa_kab | *Parvicapsula* | Parasite | 0 | F—GTCGGATGATAAGTGCATCTGATT | 1.19 |
|  | kabatai |  |  | R—ACACCACAACTCTGCCTTCCA |  |
|  |  |  |  | P—TGCGACCATCTGCACGGTACTGC |  |
| te_bry | *Tetracapsuloides* | Parasite | 2.5 | F—GCGAGATTTGTTGCATTTAAAAAG | 1.1 |
|  | *bryosalmonae* |  |  | R—GCACATGCAGTGTCCAATCG |  |
|  |  |  |  | P—CAAAATTGTGGAACCGTCCGACTACGA |  |
| pa_min | *Parvicapsula* | Parasite | 0 | F—AATAGTTGTTTGTCGTGCACTCTGT | 1.21 |
|  | minibicornis |  |  | R—CCGATAGGCTATCCAGTACCTAGTAAG |  |
|  |  |  |  | P—TGTCCACCTAGTAAGGC |  |
| sp_des | *Sphaerothecum* | Parasite | 52.5 | F—GCCGCGAGGTGTTTGC | 0.98 |
|  | destruens |  |  | R—CTCGACGCACACTCAATTAAGC |  |
|  |  |  |  | P—CGAGGGTATCCTTCCTCTCGAAATTGGC |  |
| sp_sal | *Spironucleus* | Parasite | 0 | F—AACCGGTTATTCGTGGGAAAG | 8.6 |
|  | salmonicida |  |  | R—TTAACTGCAGCAACACAATAGAATACTC |  |
|  |  |  |  | P—TGCCAGCAGCCGCGGTAATTC |  |
| ic_hof | *Ichthyophonus* | Parasite | 0 | F—GTCTGTACTGGTACGGCAGTTTC | 1.12 |
|  | hoferi |  |  | R—TCCCGAACTCAGTAGACACTCAA |  |
|  |  |  |  | P—TAAGAGCACCCACTGCCTTCGAGAAGA |  |
| na_sal | *Nanophyetus* | Fluke | 0 | F—CGATCTGCATTTGGTTCTGTAACA | 1.27 |
|  | *salmincola* |  |  | R—CCAACGCCACAATGATAGCTATAC |  |
|  |  |  |  | P—TGAGGCGTGTTTTATG |  |

Supplementary Table 2. The biomarker assays and three reference genes evaluated by qPCR on adult steelhead. For each assay the abbreviation, gene name, function, primer/probe sequences, and efficiency are presented.

| **Assay name** | **Gene name** | **Function** | **Primer and probe sequences** | **Efficiency** |
| --- | --- | --- | --- | --- |
| B2M | Beta 2-microglobulin | Immunity | F—TTTACAGCGCGGTGGAGTC | 1.08 |
|  |  |  | R—TGCCAGGGTTACGGCTGTAC |  |
|  |  |  | P—AAAGAATCTCCCCCCAAGGTGCAGG |  |
| CCL4 | Chemokine (C-C motif) ligand 4 | Immunity | F— TCTCTTCATTGCAACAATCTGCTT | 0.99 |
|  |  |  | R— ACAGCAGTCCACGGGTACCT |  |
|  |  |  | P— CTACGCAGCAGCATT |  |
| CD4 | Cluster of differentiation 4 | Immunity | F—CATTAGCCTGGGTGGTCAAT | 1.02 |
|  |  |  | R—CCCTTTCTTTGACAGGGAGA |  |
|  |  |  | P—CAGAAGAGAGAGCTGGATGTCTCCG |  |
| CD8a | T-cell surface glycoprotein CD8 alpha chain | Immunity | F—ACACCAATGACCACAACCATAGAG | 0.95 |
|  |  |  | R—GGGTCCACCTTTCCCACTTT |  |
|  |  |  | P—ACCAGCTCTACAACTGCCAAGTCGTGC |  |
| IgMs | Immunoglobulin | Immunity | F—CTTGGCTTGTTGACGATGAG | 1.07 |
|  |  |  | R—GGCTAGTGGTGTTGAATTGG |  |
|  |  |  | P—TGGAGAGAACGAGCAGTTCAGCA |  |
| IgT | Immunoglobulin tau | Immunity | F—CAACACTGACTGGAACAACAAGGT | 0.83 |
|  |  |  | R—CGTCAGCGGTTCTGTTTTGGA |  |
|  |  |  | P—AGTACAGCTGTGTGGTGCA |  |
| MHCI | Major histocompatibility complex I | Immunity | F—GCGACAGGTTTCTACCCCAGT  R—TGTCAGGTGGGAGCTTTTCTG  P—TGGTGTCCTGGCAGAAAGACGG | 1.02 |
|  |  |  |  |  |
|  |  |  |  |  |
| MHCII-B | Major histocompatibility complex IIβ | Immunity | F—TGCCATGCTGATGTGCAG | 1.06 |
|  |  |  | R—GTCCCTCAGCCAGGTCACT |  |
|  |  |  | P—CGCCTATGACTTCTACCCCAAACAAAT |  |
| TCRa | T cell receptor alpha chain | Immunity | F—ACAGCTTGCCTGGCTACAGA | 1.09 |
|  |  |  | R—TGTCCCCTTTCACTCTGGTG |  |
|  |  |  | P—CAGCGCACACAAGGCTAATTCG |  |
| ZAP7 | Tyrosine-protein kinase (ZAP-70) | Immunity | F—TCACCTCCGGACCTTTCATT | 1.02 |
|  |  |  | R—CCATGTGGGAAGCCTTTTCTT |  |
|  |  |  | P—TCTTGTATGGTTTTCCTCC |  |
| C3 | Complement component 3 | Immunity | F—ATTGGCCTGTCCAAAACACA | 0.95 |
|  |  |  | R—AGCTTCAGATCAAGGAAGAAGTTC |  |
|  |  |  | P—TGGAATCTGTGTGTCTGAACCCC |  |
| C7 | Complement factor | Immunity | F—ACCTCTGTCCAGCTCTGTGTC | 0.88 |
|  |  |  | R—GATGCTGACCACATCAAACTGC |  |
|  |  |  | P—AACTACCAGACAGTGCTG |  |
| IL-15 | Interleukin 15 | Immunity | F—TTGGATTTTGCCCTAACTGC | 0.95 |
|  |  |  | R—CTGCGCTCCAATAAACGAAT |  |
|  |  |  | P—CGAACAACGCTGATGACAGGTTTTT |  |
| IL-1B | Interleukin 1-beta | Immunity | F—AGGACAAGGACCTGCTCAACT | 0.91 |
|  |  |  | R—CCGACTCCAACTCCAACACTA |  |
|  |  |  | P—TTGCTGGAGAGTGCTGTGGAAGAA |  |
| IL-8 | Interleukin 8 | Immunity | F—GAGCGGTCAGGAGATTTGTC | 0.94 |
|  |  |  | R—TTGGCCAGCATCTTCTCAAT |  |
|  |  |  | P—ATGTCAGCGCTCCGTGGGT |  |
| MMP13 | Matrix Metallopeptidase 13 | Immunity | F—GCCAGCGGAGCAGGAA | 1.03 |
|  |  |  | R—AGTCACCTGGAGGCCAAAGA |  |
|  |  |  | P—TCAGCGAGATGCAAAG |  |
| MMP25 | Matrix Metallopeptidase 25 | Immunity | F—TGCAGTCTTTTCCCCTTGGAT | 1.04 |
|  |  |  | R—TCCACATGTACCCACACCTACAC |  |
|  |  |  | P—AGGATTGGCTGGAAGGT |  |
| PCBL | Precerebellin | Immunity | F—TGGTGTTGCTTTGCTGTTGT | 0.96 |
|  |  |  | R—GCCACTTTTGGTTTGCTCTC |  |
|  |  |  | P—ATGGTTGAGACTCAGACGGAGAGTG |  |
| SRK2 | Tyrosine-protein kinase FRK | Immunity | F—CCAACGAGAAGTTCACCATCAA | 0.94 |
|  |  |  | R—TCATGATCTCATACAGCAAGATTCC |  |
|  |  |  | P—TGTGACGTGTGGTCCT |  |
| SAA | Serum amyloid protein alpha | Immunity | F—GGGAGATGATTCAGGGTTCCA | 0.99 |
|  |  |  | R—TTACGTCCCCAGTGGTTAGC |  |
|  |  |  | P—TCGAGGACACGAGGACTCAGCA |  |
| TF | Transferrin | Immunity | F—TTCACTGCTGGAAAATGTGG | 1.02 |
|  |  |  | R—GCTGCACTGAACTGCATCAT |  |
|  |  |  | P—TGGTCCCTGTCATGGTGGAGCA |  |
| TNF | Tumour necrosis factor | Immunity | F—CCCACCATACATTGAAGCAGATT | 0.93 |
|  |  |  | R—GGATTGTATTCACCCTCTAAATGGA |  |
|  |  |  | P—CCGGCAATGCAAAA |  |
| EF2 | Eukaryotic translation elongation factor 2 | Immunity | F—AGGTCACAGCCGCCCTTAG | 1.01 |
|  |  |  | R—ACACAGTCTCTGTCTGCACACACA |  |
|  |  |  | P—CGACTGCGTCTCAGGT |  |
| IFI44A | IFN-induced protein 44-1 | VDD | F—GCTAGTGCTCTTGAGTATCTCCACAA | 0.89 |
|  |  |  | R—TCACCAGTAACTCTGTATCATCCTGTCT |  |
|  |  |  | P—AGCTGAAAGCACTTGAG |  |
| IFIT5 | Interferon-induced protein with tetratricopeptide repeats 5 | VDD | F—CCGTCAATGAGTCCCTACACATT | 0.92 |
|  |  |  | R—CACAGGCCAATTTGGTGATG |  |
|  |  |  | P—CTGTCTCCAAACTCCCA |  |
| 52Ro | 52 kDa Ro protein-2 | VDD | F—TGCACTATTGCCCAGTAACCAT | 0.98 |
|  |  |  | R—TGCAAGAGGAGATGCCAACA |  |
|  |  |  | P—AGTAGGATTCACAGAGAGTT |  |
| IRF1 | Interferon regulatory factor 1 | VDD | F—CAAACCGCAAGAGTTCCTCATT | 0.96 |
|  |  |  | R—AGTTTGGTTGTGTTTTTGCATGTAG |  |
|  |  |  | P—CTGGCGCAGCAGATA |  |
| Mx | Antiviral protein | VDD | F—AGATGATGCTGCACCTCAAGTC | 0.92 |
|  |  |  | R—CTGCAGCTGGGAAGCAAAC |  |
|  |  |  | P—ATTCCCATGGTGATCCGCTACCTGG |  |
| RSAD | Radical S-adenosyl methionine Domain-containing protein 2 | VDD | F—GGGAAATTAGTCCAATACTGCAAAC | 1.10 |
|  |  |  | R—GCCATTGCTGACAATACTGACACT |  |
|  |  |  | P—CGACCTCCAGCTCC |  |
| VIG10 | VHSV-induced protein-10 | VDD | F—GCAAACTGAGAAAACCATCAAGAA | 0.99 |
|  |  |  | R—CCGTCAGCTCCCTCTGCAT |  |
|  |  |  | P—TGTGGAGAAGTTGCAGGC |  |
| DEXH | [DEXH box helicase, DNA ligase-associated](http://www.ebi.ac.uk/interpro/entry/IPR026362) | VDD | F—CCATAAGGAGGGTGTCTACAATAAGAT | 0.90 |
|  |  |  | R—CTCTCCCCCTTCAGCTTCTGT |  |
|  |  |  | P—TGGCGCGCTACGTG |  |
| GAL3 | Galectin-3-binding protein precursor | VDD | F—TTGTAGCGCCTGTTGTAATCATATC | 0.95 |
|  |  |  | R—TACACTGCTGAGGCCATGGA |  |
|  |  |  | P—CTTGGCGTGGTGGC |  |
| NFX | Zinc finger NFX1-type | VDD | F—CCACTTGCCAGAGCATGGT | 0.95 |
|  |  |  | R—CGTAACTGCCCAGAGTGCAAT |  |
|  |  |  | P—TGCTCCACCGATCG |  |
| STAT1 | Signal transducer and activator of transcription 1-alpha/beta | VDD | F—TGTCACCGTCTCAGACAGATCTG | 0.85 |
|  |  |  | R—TGTTGGTCTCTGTAAGGCAACGT |  |
|  |  |  | P—AGTTGCTGAAAACCGG |  |
| VAR1 | Mitochondrial ribosomal protein (VAR1) | VDD | F—CCACCTGAGGTACTGAAGATAAGACA | 0.99 |
|  |  |  | R—TTAAGTCCTCCTTCCTCATCTGGTA |  |
|  |  |  | P—TCTACCAGGCCTTAAAG |  |
| HBA | Hemoglobin subunit alpha | Stress | F—GCCCTGGCTGACAAATACAGA | 1.12 |
|  |  |  | R—GAGCAGGAACTGGAGTCCAATG |  |
|  |  |  | P—ACCATCATGAAAGTCC |  |
| CA4 | Carbonic anhydrase 4 | Stress | F—GGTCATTTTGGTTTTGTACACAGTCT | 0.92 |
|  |  |  | R—CCTAGATATAGCTATCCACGTACTCACCTA |  |
|  |  |  | P—TGATACGTGGTATAGAAAAG |  |
| HIF1A_3 | Hypoxia-inducible factor 1-alpha | Stress | F—CACTACAACTTCTCCTCACTCACTCTGT | 1.11 |
|  |  |  | R—AGCAGCCAAACTATAAGATCACTGATAC |  |
|  |  |  | P—CTGCCCCTTTATTTGTCTC |  |
| HIF1A_7 | Hypoxia-inducible factor 1-alpha | Stress | F—TGGCAAATCTGCCTACGAATT | 0.99 |
|  |  |  | R—GCAGGCTCTTGGTCACATGA |  |
|  |  |  | P—ATCATGCCCTGGACTC |  |
| HSC70 | Heat shock cognate 70 | Stress | F—GGGTCACACAGAAGCCAAAAG | 0.88 |
|  |  |  | R—GCGCTCTATAGCGTTGATTGGT |  |
|  |  |  | P—AGACCAAGCCTAAACTA |  |
| HSP90a | Heat shock protein 90-alpha | Stress | F—AGTACCCTGTTGCACTGAGTTTTAAA | 0.88 |
|  |  |  | R—GAATGTTTCATTTCCCATTGTTCA |  |
|  |  |  | P—ATTGGACTGGTAGATGTGT |  |
| HSP90 (alike) | Heat shock protein 90-alpha (alike) | Stress | F—TTGGATGACCCTCAGACACACT | 1.05 |
|  |  |  | R—CGTCAATACCCAGGCCTAGCT |  |
|  |  |  | P—CCGAATCTACCGGATGAT |  |
| MAP3K | Mitogen-activated protein kinase kinase kinase 14è Mapk14 | Stress | F—GCTCCCTGGGTTCATGGAT | 1.08 |
|  |  |  | R—GCCTCCCTTCAGCAGAGACA |  |
|  |  |  | P—CCAGCAATAGCTTATG |  |
| PARK7 | Parkinson disease (autosomal recessive, early onset) 7; SP22 | Stress | F—ACTGCAAGCAGCATGATCAACT | 1.02 |
|  |  |  | R—TTGGCCTGTGTATCATAATGAACA |  |
|  |  |  | P—CCCCACCTACTCAGC |  |
| SEPW1 | Selenoprotein W | Stress | F—TGAGGATGAATTCCCAGGTGAT | 1.12 |
|  |  |  | R—AAACCACCCAGAGGTTGAAGGT |  |
|  |  |  | P—TTGAGATTACTGGTGAAAGC |  |
| SERPIN | serpin H1-Precursor (heat shock protein 47) | Stress | F—ACTATGACCACTCGAAGATCAACCT | 0.86 |
|  |  |  | R—CCCATTCGTTGATGGAGTTCA |  |
|  |  |  | P—AGGGACAAGAGGAGC |  |
| COMMD7 | COMM domain-containing protein 7 | MRS | F—CAAAGCCAGTATGGACTGTTTCAG  R—TTGTTTTCTGCTGCCCCTCTA  P—ACCTGATCGCCAGTAGCATGAGCATGTAC | 0.89 |
|  |  |  |  |  |
|  |  |  |  |  |
| FYB | FYN-binding protein | MRS | F—TGCAGATGAGCTTGTTGTCTACAG | 1.01 |
|  |  |  | R—GCAGTAAAGATCTGCCGTTGAGA |  |
|  |  |  | P—CTCAACGATGACATCCACAGTCTCCCC |  |
| HTA | Histone acetyltransferase | MRS | F—CTTGTAACAGTTCGACATGGCTTATT | 0.84 |
|  |  |  | R—TGGTGAAGCATTTCTGTATGTCAA |  |
|  |  |  | P—TCTGTACTGAGCATCCCCGCACATTACA |  |
| KRT8 | Keratin, type II cytoskeletal 8 | MRS | F—CGATTGAGCGGCTGGATAA | 1.04 |
|  |  |  | R—GCATTGTTTACCTTTGACTTGAATTG |  |
|  |  |  | P—CCCCCTTCTCTACTCTCTTGCTCACCATTC |  |
| SCG | Secretogranin II | MRS | F—GGATGTGAAGAATCCAACACTGAT | 0.87 |
|  |  |  | R—ACACCACTTCAAACTAGCCATACATT |  |
|  |  |  | P—CGGCTGTATGTGCACTG |  |
| NKAa1a | Na/K ATPase α-1a (freshwater) | Osmoregulation | F—TGGAATCAAGGTTATCATGGTCACT | 1.00 |
|  |  |  | R—CCCACACCCTTGGCAATG |  |
|  |  |  | P—ATCATCCCATCACTGCGA |  |
| NKAa1b | Na/K ATPase α-1b (saltwater) | Osmoregulation | F—GCCTGGTGAAGAATCTTGAAGCT | 1.13 |
|  |  |  | R—GAGTCAGGGTTCCGGTCTTG |  |
|  |  |  | P—CCTCCACCATTTGCTCA |  |
| ALDOA | Aldolase A, fructose-bisphosphate | Metabolism | F—CGTGATTCAGTGTTGTCATCTTGA | 0.94 |
|  |  |  | R—TTCCTCCAGTGTTTTTTTCAGTCA |  |
|  |  |  | P—AAGTACATGTGCCTTCTT |  |
| COX6B1 | Cytochrome C oxidase | Metabolism | F—GCCCCGTGTGACTGGTATAAG | 0.86 |
|  |  |  | R—TCGTCCCATTTCTGGATCCA |  |
|  |  |  | P—TCTACAAATCACTGTGCCC |  |
| IDH3b | Isocitrate dehydrogenase 3 (NAD+)-beta | Metabolism | F—TCGTGTTTGGCTGTTCAGTCA | 0.80 |
|  |  |  | R—AGTGGCTTGTTCGTTTGCAA |  |
|  |  |  | P—CAAAGCTCTTTCATCATT |  |
| LDHB | Lactate dehydrogenase B | Metabolism | F—GTCACTGCTCCCATTTTACACTCTAG | 0.85 |
|  |  |  | R—CCCAAACTCCCTCCCAGATAAC |  |
|  |  |  | P—CTGTTCTTAGCTTCCC |  |
| MPDU1 | Mannose-P-Dolichol Utilization Defect 1 Protein (alike) | Metabolism | F—TGCTTGACCCCTTGATTATAGCTA | 1.05 |
|  |  |  | R—GACCATAATCTAGAATGAAAACGCATT |  |
|  |  |  | P—CTTCCTGGTTGTGTTCTG |  |
| PGK3 | Phosphoglycerate kinase | Metabolism | F—GGCAAAGTGCTCCCTAAGTTTC | 1.12 |
|  |  |  | R—TAGAGAGCAGGGCTGGTGCTA |  |
|  |  |  | P—CACCCTGCGCTTGT |  |
| Sema4ab | Sema domain, immunoglobulin domain, transmembrane domain, and short cytoplasmic domain, (semaphorin) 4Ab | Reference | F—GTCAAGACTGGAGGCTCAGAG | 0.98 |
|  |  |  | R—GATCAAGCCCCAGAAGTGTTTG |  |
|  |  |  | P—AAGGTGATTCCCTCGCCGTCCGA |  |
| COIL-P84-2 | Coiled-coil domain-containing protein 84 | Reference | F—GCTCATTTGAGGAGAAGGAGGATG | 0.98 |
|  |  |  | R—CTGGCGATGCTGTTCCTGAG |  |
|  |  |  | P—TTATCAAGCAGCAAGCC |  |
| MRPL40 | 39S ribosomal protein L40, mitochondrial precursor | Reference | F—CCCAGTATGAGGCACCTGAAGG | 0.94 |
|  |  |  | R—GTTAATGCTGCCACCCTCTCAC |  |
|  |  |  | P—ACAACAACATCACCA |  |
| ACTB | Beta-actin | Excluded | F—GAAATCGCCGCACTGGTT | 1.38 |
|  |  |  | R—CGGCGAATCCGGCTTT |  |
|  |  |  | P—TTGACAACGGATCCGGT |  |
| C1Qc | complement C1q subcomponent subunit C | Excluded | F—CGCCGGTGAGTGGAATCTA | 0.69 |
|  |  |  | R—CTTCTCCATCATGTGGTGTGCTA |  |
|  |  |  | P—ACCTCCAAACATAGAAGAG |  |
| Cd83 | Cluster of Differentiation 83 | Excluded | F—GTGGCGGCATTGCTGATATT | Failed |
|  |  |  | R—CTTGTGGATACTTCTTACTCCTTTGCA |  |
|  |  |  | P—CACCATCAGCTATGTCATCC |  |
| CIRBP | Cold-inducible RNA-binding protein | Excluded | F—TGATTGACTGTTTTGCCAACTGA | Failed |
|  |  |  | R—TCAGACCTTTGTGTGCATTTACCT |  |
|  |  |  | P—ATGGTGATGAGCCTGAAT |  |
| FK506 | FK506-binding protein 10 precursor | Excluded | F—ACTATGAGAATGCCCCCATCAC | Failed |
|  |  |  | R—CTCGTCCAGACCCTCAATCAC |  |
|  |  |  | P—CCTGGGAGCCAACAA |  |
| Glut2 | Solute carrier family 2, facilitated glucose transporter member 2-like | Excluded | F—GGAACCTTACATCAACTGGCTACA | Failed |
|  |  |  | R—GCAGTGGCCAGTAGTAGTCATTACC |  |
|  |  |  | P—CTGGTATACTACTGAGTCAGG |  |
| Hep | Hepcidin | Excluded | F—GAGGAGGTTGGAAGCATTGA | 1.28 |
|  |  |  | R—TGACGCTTGAACCTGAAATG |  |
|  |  |  | P—AGTCCAGTTGGGGAACATCAACAG |  |
| HERC6 | Probable E3 ubiquitin-protein ligase | Excluded | F—AGGGACAACTTGGTAGACAGAAGAA | 1.24 |
|  |  |  | R—TGACGCACACACAGCTACAGAGT |  |
|  |  |  | P—CAGTGGTCTCTGTGGCT |  |
| HIF1A-6 | Hypoxia-inducible factor 1-alpha-like | Excluded | F—AGAGGAGGCAGTGCTGTATTCAA | Failed |
|  |  |  | R—GGGACAAGGCCCTCCAAT |  |
|  |  |  | P—AGGGCCCTGACCATG |  |
| IGFBP1 | Insulin-like growth factor binding protein-1 | Excluded | F—AGATAACCAGCTCTCAGCAGGAA | Failed |
|  |  |  | R—ATGTTTGTCACAGTTGGGTAGGTAGA |  |
|  |  |  | P—TAGGAGAGAAGTTCACCAAC |  |
| IL-11 | Interleukin 11 | Excluded | F—GCAATCTCTTGCCTCCACTC | 0.79 |
|  |  |  | R—TTGTCACGTGCTCCAGTTTC |  |
|  |  |  | P—TCGCGGAGTGTGAAAGGCAGA |  |
| JUN | AP-1 Transcription Factor Subunit | Excluded | F—TTGTTGCTGGTGAGAAAACTCAGT | Failed |
|  |  |  | R—CCTGTTGCCCTATGAATTGTCTAGT |  |
|  |  |  | P—AGACTTGGGCTATTTAC |  |
| LdhaL | L-lactate dehydrogenase A chain-like | Excluded | F—TTTGTTTAGTGTGTGCGAGAGTTG | Failed |
|  |  |  | R—TCCGTGCACTTACGGTTAGTTTT |  |
|  |  |  | P—CCAGAGCCATTCAGT |  |
| SHOP21 | Salmon hyperosmotic protein 21 | Excluded | F—GCGGTAGTGGAGTCAGTTGGA | 1.29 |
|  |  |  | R—GCTGCTGACGTCTCACATCAC |  |
|  |  |  | P—CCTGTTGATGCTCAAGG |  |
| TCRb | T cell receptor beta chain | Excluded | F—TCACCAGCAGACTGAGAGTCC | 4.02 |
|  |  |  | R—AAGCTGACAATGCAGGTGAATC |  |
|  |  |  | P—CCAATGAATGGCACAAACCAGAGAA |  |
| VIG4 | VHSV-inducible protein-4 | Excluded | F—TGGCTTCCCACATTGCAA | 0.80 |
|  |  |  | R—CCTCCTCCCCCCTGCAT |  |
|  |  |  | P—AGATGGAGACAGGAATG |  |
